# Structure/function studies of the NAD^+^-dependent DNA ligase from the poly-extremophile *Deinococcus radiodurans* reveal importance of the BRCT domain for DNA binding

**DOI:** 10.1007/s00792-023-01309-z

**Published:** 2023-09-15

**Authors:** Andreia Fernandes, Adele Williamson, Pedro M. Matias, Elin Moe

**Affiliations:** 1 Instituto de Tecnologia Química e Biológica António Xavier, Universidade NOVA de Lisboa, Oeiras, Portugal; 2 Department of Chemistry, UiT—The Arctic University of Norway, Tromsø, Norway; 3 School of Science, University of Waikato, Hamilton, New Zealand; 4 Institute of Experimental and Technological Biology (IBET), Oeiras, Portugal

**Keywords:** DNA ligase A, BRCT, DNA nick-joining, Protein–DNA binding, X-ray crystallography

## Abstract

Bacterial NAD^+^-dependent DNA ligases (LigAs) are enzymes involved in replication, recombination, and DNA-repair processes by catalyzing the formation of phosphodiester bonds in the backbone of DNA. These multidomain proteins exhibit four modular domains, that are highly conserved across species, with the BRCT (breast cancer type 1 C-terminus) domain on the C-terminus of the enzyme. In this study, we expressed and purified both recombinant full-length and a C-terminally truncated LigA from *Deinococcus radiodurans* (DrLigA and DrLigA∆BRCT) and characterized them using biochemical and X-ray crystallography techniques. Using seeds of DrLigA spherulites, we obtained ≤ 100 µm plate crystals of DrLigA∆BRCT. The crystal structure of the truncated protein was obtained at 3.4 Å resolution, revealing DrLigA∆BRCT in a non-adenylated state. Using molecular beacon-based activity assays, we demonstrated that DNA ligation via nick sealing remains unaffected in the truncated DrLigA∆BRCT. However, DNA-binding assays revealed a reduction in the affinity of DrLigA∆BRCT for dsDNA. Thus, we conclude that the flexible BRCT domain, while not critical for DNA nick-joining, plays a role in the DNA binding process, which may be a conserved function of the BRCT domain in LigA-type DNA ligases.

**Supplementary Information:**

The online version contains supplementary material available at 10.1007/s00792-023-01309-z.

## Introduction

DNA ligases are crucial enzymes to maintain genome integrity by joining 3′-OH group and 5′-PO_4_ termini to form a phosphodiester bond in double-stranded DNA molecules (Lehman 1974). They are members of the large and diverse superfamily of nucleotidyltransferases, which act by carrying out three nucleotidyl transfer steps. First, in the autoadenylation step, an AMP group derived from the cofactor is transferred to a conserved lysine residue in the enzyme active site. Then, the AMP is transferred from the enzyme to the 5′-PO_4_ group of the DNA nick, activating the 5′-PO_4_ for nucleophilic attack by the 3′-OH of the DNA nick. Thus, in the final step, a 3′-5′ phosphodiester bond is formed, and AMP is released (Lehman 1974; Tomkinson et al. 2006; Pascal 2008; Williamson and Leiros 2020).

Depending on the cofactor, ligases can be categorized into two classes: ATP-dependent DNA ligases that are present in Archaea, viruses and Eukarya; and NAD^+^-dependent DNA ligases that are found almost exclusively in Bacteria. However, additional genes encoding ATP-dependent DNA ligases have been identified in some bacterial genomes (Timson et al. 2000; Wilkinson et al. 2001). One of these organisms is *Deinococcus radiodurans*, a bacterium that is extremely resistant to radiation and DNA damage. *D. radiodurans* encodes an NAD^+^-dependent DNA ligase (DrLigA) and an ATP-dependent DNA ligase (DrLigB) (White et al. 1999). The DrLigB gene is included in a three-gene operon that is inducible when cells are exposed to gamma radiation (Liu et al. 2003). DrLigB has been characterized by Kota et al. (2010) who demonstrated that DrLigB possesses DNA end-joining activity but requires other deinococcal proteins, namely PprA (DrB0099) and protein DrB0098 for its function. On the contrary, DrLigA possesses DNA ligation activity with different DNA substrates, requiring only the cofactor NAD^+^ and divalent metals to be active (Blasius et al. 2007; Le et al. 2008). As with other DNA ligases, DrLigA activity is abolished by the mutation of the conserved lysine 128 to alanine, where the AMP group binds, adenylation is a requirement for enzyme activity (Blasius et al. 2007). Although both deinococcal ligases are involved in DNA repair, their functional redundancy has not been studied. It has been demonstrated that NAD^+^-dependent DNA ligases (LigA) are essential, even in bacteria which have multiple ligases (Petit and Ehrlich 2000). Therefore, we are interested in understanding the role and structural features of DrLigA as a DNA-repair enzyme.

**Fig. 1:**
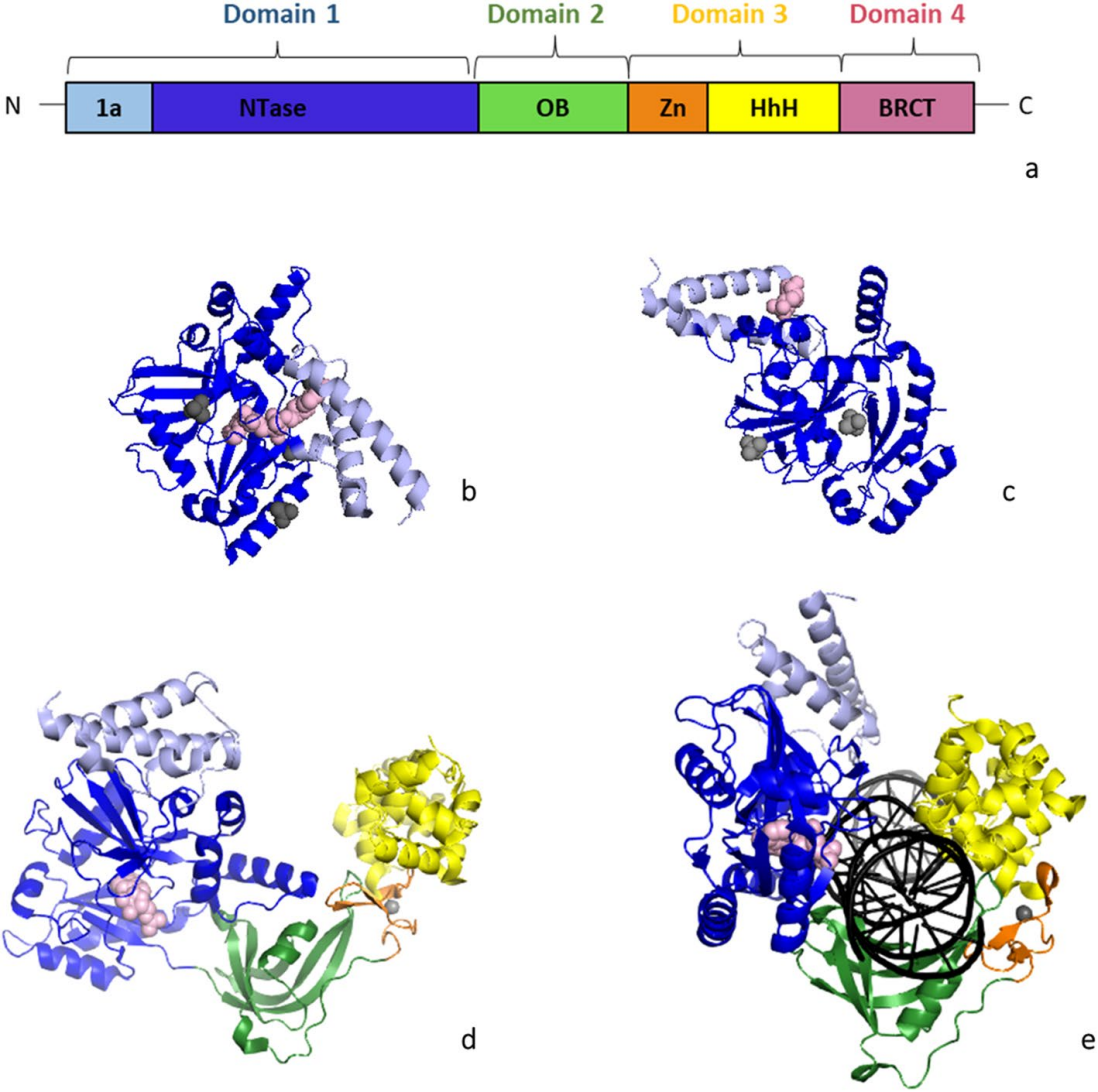
Representation of the modular organization and structures of bacterial DNA ligases A **a** identification of the domains and subdomains: Domain 1 with subdomain 1a (light blue) and 1b/NTase (blue), Domain 2/OB (green), Domain 3 with subdomain 3a/Zn (orange) and 3b/HhH (yellow), and Domain 4/BRCT (purple). *NTase* nucleotidyl transferase, *OB* oligonucleotide-binding, *Zn* zinc finger, *HhH* helix–hairpin–helix, *BRCT* breast cancer type 1 C-terminus. **b** *E. faecalis* truncated LigA (PDB ID: 1tae). **c** *E. faecalis* truncated LigA (PDB: 1ta8), **d** *T. filiformis* apo-LigA (PDB ID: 1dgs). **e** *E. coli* LigA–DNA complex (PDB ID: 2owo). Metals (gray) and cofactor (light pink) are shown as spheres, and DNA is colored black

The first structure of a LigA was the N-terminal domain structure from *Bacillus stearothermophilus* (Singleton et al. 1999), showing that this domain shares structural resemblance to the adenylation core of the ATP-ligases’ counterparts. Additionally, crystal structures of truncated LigA proteins bound to NAD^+^, AMP, or NMN, such as *Enterococcus faecalis* (EfLigA) (Gajiwala and Pinko 2004) and *Mycobacterium tuberculosis* (Srivastava et al. 2005) structures, also revealed information about the adenylation core. EfLigA structures have revealed details for the recognition of NAD^+^ and how the almost 180° turn of two helices of the N-terminal is important for the enzyme’s adenylation (Fig. 1b, c) (Gajiwala and Pinko 2004). Then, the first full-length LigA structure, the *Thermus filiformis* LigA (ThLigA) structure, revealed the full modular organization of these enzymes, consisting of four domains in a circular rearrangement (Lee et al. 2000). It contains a: (i) N-terminal domain 1 with the nucleotidyltransferase (NTase) subdomain; (ii) oligo-binding (OB) fold domain 2; (iii) domain 3 which includes zinc finger motif and helix–hairpin–helix (HhH) motifs; and iv) C-terminal domain 4 (Fig. 1a, d). Further details of an enzyme bound to adenylated nicked double-stranded DNA (dsDNA) were shown, the structure of *E. coli* LigA–DNA complex captured a state prior to ligation (Nandakumar et al. 2007). This structure showed that EcligA envelopes the DNA as a clamp, for that it is essential the almost 180° rotation of domain 2 (Fig. 1e). Taken together, all these structures have provided insights into the mechanisms of adenylation, ligation, and DNA binding by NAD^+^-dependent DNA ligases.

DrLigA is predicted to contain the common modular architecture seen across the bacterial ligases. The 700-residue protein consists of: (i) domain 1 which contains a N-terminal subdomain 1a and NTase subdomain 1b; (ii) domain 2 which is an oligo-binding (OB) fold domain; (iii) domain 3 which includes a zinc finger motif 3a and helix–hairpin–helix (HhH) subdomain 3b; and (iv) C-terminal domain 4 which comprises one breast cancer type 1 C-terminus (BRCT) domain. Regarding their functions, N-terminal subdomain 1a confers the enzyme’s NAD^+^ specificity, and the NTase subdomain 1b contains the catalytic site. It is considered for NAD^+^-dependent ligases as well as for ATP-dependent ligases that the catalytic core includes the NTase subdomain 1b and the OB-fold domain 2. These two domains form the minimal catalytic unit. While the other domains, domain 3 and domain 4 as well as domain 2 are involved in the DNA-binding process. BRCT domain 4 function is still not well known, BRCT may influence DNA binding or may be involved in the DNA targeting (Pergolizzi et al. 2016).

Here, we report the cloning, expression, purification, and characterization of both full-length DrLigA and a C-terminally truncated protein (DrLigAΔBRCT). We determined the crystal structure of the non-adenylated apo-DrLigAΔBRCT to a resolution of 3.4 Å. Activity measurements demonstrated that the ligation activity of DrLigAΔBRCT was preserved, but its affinity to dsDNA diminished in comparison to the full-length protein. We thus conclude that the C-terminal BRCT domain of DrLigA is important for the protein binding to DNA.

## Materials and methods

### Cloning, protein expression, and purification

The gene-encoding DrLigA (DR_2069) and DrLigAΔBRCT were amplified according to the PCR reactions described by Fernandes et al. (2021). For DrLigA amplification, the primers FPDrLigA and RPDrLigA were used in the first PCR reaction. A second reaction was performed using primers FDRalle and RPDrLigA (Supplementary Table S1). The DrLigAΔBRCT was generated using primer RPbrct for amplification of the gene instead of primer RPDrLigA (Supplementary Table S1). Both constructs contain a His_6_-tag and TEV cleavage site at the N-terminus. The genes were cloned into the expression vector pDest14 according to the GATEWAY cloning system (GE Healthcare) guidelines. Protein expression experiments were performed according to the small-, medium-, and large-scale expression protocols as described previously for other *D. radiodurans* enzymes (Fernandes et al. 2021). The best conditions for expressing DrLigA were at 37 °C for 3 h with the *E. coli* strain BL21(DE3)* pRARE2. Therefore, in large-scale expression of the DrLigA and DrLigAΔBRCT, proteins were expressed according to these conditions and expression was induced with the addition of 0.5 mM of isopropyl β-D-1-thiogalactopyranoside. The initial purification procedure of both full-length and truncated proteins followed three steps: (i) immobilization on metal ion affinity chromatography (IMAC) with a 5 mL HisTrap HP column, (ii) followed by TEV protease cleavage of the His_6_-tag and a (iii) subsequent second IMAC step to remove the tag (Fernandes et al. 2021). The final purification step was performed using a 1 mL HiTrap Heparin HP column. The column was pre-equilibrated with buffer 2 (20 mM Bis–Tris pH 6.0, 150 mM NaCl). For elution, a gradient from 150 to 1000 mM NaCl in buffer 2 was applied. Fractions containing protein were identified using 10% Tris–glycine-SDS-PAGE. The final protein pool was desalted in buffer 2 using a PD-10 desalting column (Cytiva) and then concentrated using Amicon Ultra centrifugal filters (Merck Millipore) with a 30 kDa MW cutoff. Protein samples were flash frozen in liquid nitrogen and stored at − 80 °C.

### Thermal shift assay

Thermal shift assays were performed with SYPRO orange dye (Invitrogen) (Ericsson et al. 2006). A 20 µL mix was prepared, containing buffer (buffer screen description is provided in Supplementary Table S2), dye to a final concentration of 10 × and protein to a final concentration of 5 μg. For the additives’ experiments, an 18 µL mix was prepared, and 2 µL solution of additive (300 µM NAD^+^, 1 mM Mg^2+^, 1 mM Mn^2+^, or 1 mM Zn^2+^) was added. Fluorescence was measured each minute over a range of temperatures from 25 to 90 °C, as previously described by Fernandes et al. (2021). The peak minimum of the first derivative of Relative Fluorescence Units over temperature was determined as the melting temperature (T_m_).

### Molecular beacon activity assay

The phosphorylated 5′-nick molecular beacon was prepared by mixing the oligos Molecular Beacon L, Nick 1 and Nick 2 according to (Tang et al. 2003) (Table 1) and preincubating them at 30 °C for 30 min. DNA ligation by nick sealing was performed with 0.4 µM of phosphorylated 5′-nick molecular beacon, 5 mM dithiothreitol, 200 μg.mL^−1^ BSA, 2% glycerol, 1 mM MnSO_4_, NAD^+^ (variable concentration), NaCl (variable concentration), and 20 mM buffer (Tris–HCl for pH ≥ 7.0, Bis–Tris for pH ≤ 7.0). The 100 µL reactions were initiated by adding 170 nM of enzyme (for the NAD^+^ concentration experiments) or 330 nM (for the NaCl concentration and pH experiments). TAMRA fluorescence, triggered by ligation-induced opening of the beacon, was measured by exciting at 525 nm and recording emission at 578 nm. The measurements were carried out on a microplate reader (SpectraMax M2, for the NAD^+^ concentration experiments, or TECAN SPARK 10 M in case of the NaCl concentration and pH experiments) at 30 °C for 30 min using black non-binding surface 96-well plates (Corning). Rates of DNA ligation activity were calculated from the increase in fluorescence as a function of time, using the linear portion of the reaction (initial velocity).

**Table 1:** DNA substrates’ sequences for molecular beacon

Name	Sequence 5′-> 3′
Molecular beacon L	*CGTTGATGGTTCCACTTCTCGTGCGTTCAACG Δ
Nick 1	CGCACGAGA
Nick 2	p AGTGGAACC

*TAMRA (Carboxytetramethylrhodamine), Δ Dabcyl, p Phosphate

### Gel-shift assay

Different DNA substrates were used to measure DNA binding by gel shift: (i) single nicked 40 nt dsDNA (Template T, N1, and N2, Table 2); (ii) 1 nt gapped 40 nt dsDNA (Template T, G1, and G2, Table 2); and (iii) single nick with 5′-flap 40 nt dsDNA (Template T, F1, and N2, Table 2). Each substrate mix was prepared in 10 mM Tris–HCl pH 8.5, to a final concentration of 0.5 µM of the labelled strand, and 2.5 µM of the unlabeled oligos. The substrate annealing was performed by heating the mixture at 95 °C for 5 min and then gradually cooling overnight. The protein–DNA solution was prepared in buffer 3 (20 mM Bis–Tris pH 6.0, 5 mM MgSO_4_, 5 mM MnSO_4_, 300 µM NAD^+^, and 25% glycerol) by adding 100 nM of dsDNA substrate and varying concentrations of protein. The protein-DNA solution was incubated at room temperature for 15 min, then each sample was loaded onto a 6% polyacrylamide gel mixed with 2.5% glycerol, which was run at 4 °C in 1 × TBE buffer. The gel was scanned for FAM fluoresce with the FLA-5100 (FujiFilm) imager.

**Table 2:** DNA substrates’ sequences for DNA-binding assays

Name	Sequence 5′-> 3′
Template T	ATTGAGTGGACAAAGTATCGTAGGGTAGTATTGGTGGATA
N1	CGATACTTTGTCCACTCAAT*
N2	TATCCACCAATACTACCCTA
G1	*TATCCACCAATACTACCCTAC
G2	ATACTTTGTCCACT
F1	*TAACGCTGGGCGATACTTTGTCCACTCAAT

*FAM (6-carboxyfluorescein)

### DrLigA∆BRCT crystallization

The DrLigA (final concentration of 12 mg/mL) and DrLigA∆BRCT (final concentration of 9 mg/mL) samples in buffer 2 were pre-incubated with 5 mM MnSO_4_ and 300 µM NAD^+^ for 1 h on ice prior to crystallization experiments. Matrix screening experiments with DrLigA were performed using the crystallization robot Mosquito LCP (SPT Labtech), and different commercial screens (listed in Supplementary Table S3). The best hit was obtained at 22 °C from solution A (1 M sodium acetate trihydrate, 0.1 M sodium HEPES pH 7.5, and 0.05 M cadmium sulfate 8/3 hydrate). Under these conditions, DrLigA spherulites appeared after 5–7 days in 200–300 nL drops at different ratios of protein:reservoir (100:200, 100:100, and 200:100 nL). These DrLigA spherulites were used to prepare seed stock A according to the seed-bead method (D’Arcy et al. 2007). Then, microseed matrix screening experiments were carried out by establishing drops that consisted of 100:70:30 nL mix of protein: reservoir: seeds A. Different DrLigA crystals were obtained, but due to their poor quality, further crystallization experiments were carried out with the truncated DrLigA∆BRCT. Several DrLigA∆BRCT crystals were obtained, including the thin plate crystals that grew for 1–3 days at 22 °C in solution B (0.2 M magnesium acetate tetrahydrate, 0.1 M sodium cacodylate pH 6.5, 20% PEG 8000). Crystal optimization experiments were performed by hanging drop vapor diffusion in 24-well MD3-11 plates (Molecular Dimensions) with 0.5 mL of reservoir solution. The plate crystals were optimized via additive screening experiments using matrix Hampton HR-138 (Hampton Research). Additive trimethylamine hydrochloride (final concentration of 0.01 M) was premixed with reservoir solution B, according to Hampton recommendations. Drops were prepared by mixing 1.0 μL DrLigA∆BRCT, 0.7 μL reservoir solution B, and 0.3 μL seed stock A.

### X-ray data collection, data analysis, and structure determination of DrLigA∆BRCT

The DrLigA∆BRCT crystals were transferred to a cryoprotectant consisting of solution B supplemented with 25% glycerol, followed by flash-freezing in liquid nitrogen. X-ray diffraction data were collected at the ALBA beamline XALOC (Juanhuix et al. 2014) to 3.4 Å resolution. The data were processed with autoPROC (Vonrhein et al. 2011), which uses XDS (Kabsch 2010) for integration, scaling, and merging data. STARANISO (Tickle et al. 2018) was used for anisotropic resolution cut-off. The structure was solved by molecular replacement (MR) with PHASER (McCoy et al. 2007) in the CCP4 suite (Winn et al. 2011). The phasing model was predicted by AlphaFold2 (Jumper et al. 2021) using the sequence of DrLigA∆BRCT (residues 1–600) through Google Colab (https://colab.research.google.com/github/sokrypton/ColabFold/blob/main/beta/AlphaFold2_advanced.ipynb). For MR, we used fragment 1b (residues 79–325), fragment 2 (residues 326–429), and fragment 3 (residues 437–600) as separate phasing models. Model building was performed by combining results obtained with Buccaneer (Cowtan 2006) and ModelCraft (Bond and Cowtan 2020). The built model was corrected and completed with COOT (Emsley et al. 2010). Initial refinement was carried out with REFMAC (Kovalevskiy et al. 2018), and subsequent structure refinements were performed using PHENIX (Liebschner et al. 2019). The final model was checked and corrected with COOT against σ_A_-weighted 2 |F_o_|–|F_c_| and |F_c_|–|F_o_| electron density maps. Four Zn^2+^ and two Mn^2+^ cations were added manually in COOT. Hydrogen atoms were added in calculated positions with the PHENIX.READYSET tool. Isotropic displacement parameters (ADPs) were refined for all non-hydrogen atoms. TLS rigid body refinement of anisotropic ADPs was performed in the final refinement cycles using three rigid body groups for the protein chain, estimated by PHENIX from a prior fully isotropic refinement. Relative X-ray/stereochemistry and X-ray/ADP weights were optimized to reduce the gap between R-work and R-free. The final R-work/R-free was 0.214/0.273, with a maximum-likelihood estimation of the overall coordinate error of 0.51 Å. The final model was analyzed with MolProbity (Williams et al. 2018): there are six outliers in the Ramachandran φ, Φ plot, and eight side-chain rotamer outliers, all located in regions of poor electron density. Complete information about data collection, data processing, and refinement is presented in Table 3. Figures were prepared with PyMOL (https://pymol.org/2/). The electrostatic surface potential was visualized using the APBS2 plugin (Jurrus et al. 2018) in PyMOL with default settings.

**Table 3:** Data collection, data processing, and refinement statistics

Data collection and processing	
Beamline	ALBA XALOC BL13
Detector	PILATUS 6 M
Space group	P 2_1_ 2 2_1_
Wavelength (Å)	0.97926
Cell parameters	
a, b, c	70.84, 73.43, 149.94
α, β, γ	90.0, 90.0, 90.0
Resolution limits of ellipsoid fitted to resolution cut-off surface (Å)	3.52, 3.95, 3.35
Resolution limits (Å)	149.94–3.36 (3.65–3.36)
R_merge_^a^	0.218 (1.552)
R_pim_^a^	0.120 (0.739)
Total number of observations	29,515 (2024)
Total number unique	7848 (393)
Mean < I/σ(I) >	5.7 (1.3)
Completeness (spherical)	67.1 (15.8)
Completeness (ellipsoidal)	81.2 (48.9)
Multiplicity	3.8 (5.2)
CC^1/2^	0.991 (0.422)
Wilson B-value (Å^2^)	110.08
Z^b^	1
Estimated V_M_^b^	2.98
Estimated solvent content (%)^b^	58.7
Refinement	
Resolution range (Å)	74.97–3.36 (3.84–3.36)
R-work^c^	0.215 (0.303)
R-free^c^	0.275 (0.456)
ML coordinate error (Å)^d^	0.50
Nr. protein non-hydrogen atoms	3926
Nr. metal ions	6
Regions omitted (a.a)	1–80, 594–600
R.m.s. deviations from ideal values	
Bond lengths (Å)	0.0020
Bond angles (º)	0.478
Planar groups (Å)	0.052
Ramachandran favored (%)^e^	91.0
Ramachandran outliers (%)^e^	1.2
Rotamer outliers (%)^e^	1.97
C^β^ outliers^e^	0
Clash score^e^	5.36

^a^R_merge_ = ∑_hkl_ ∑_i_ | *I*_i_(hkl)—< *I*(hkl) >|/∑_hkl_ ∑_i_ *I*_i_(hkl); R_pim_ = ∑_hkl_ [1/n − 1]^1/2^ ∑_i_ | *I*_i_(hkl)—< *I*(hkl) >|/∑_hkl_ ∑_i_ *I*_i_(hkl); where for each unique Bragg reflection with indices (hkl), *l*_i_ is the *i*th observation of its intensity and n its multiplicity

^b^Z = number of molecules in the asymmetric unit. Estimation of V_M_ and solvent content is based on Matthews Probabilities (Winn et al. 2011)

^c^Rwork = ∑_hkl_ | | F_obs_(hkl) |—| F_calc_(hkl) | |/∑_hkl_ | F_obs_(hkl) |; Rfree is calculated as above from a random sample containing 5% of the total number of independent reflections measured

^d^Maximum-likelihood error estimated by PHENIX

^e^Calculated with MolProbity

## Results and discussion

### Protein purification and buffer optimization

DrLigA and DrLigA∆BRCT were expressed recombinantly in *E. coli* BL21 (DE3)* pRARE2 and purified to an apparent purity above 95% with final yields of 1.9 mg/L and 1.2 mg/L of culture, respectively (Supplementary Fig. S1a, b). After the initial purification, the protein was stored in 50 mM Tris–HCl pH 7.5 with 150 mM NaCl (buffer 1).

**Fig. 2:**
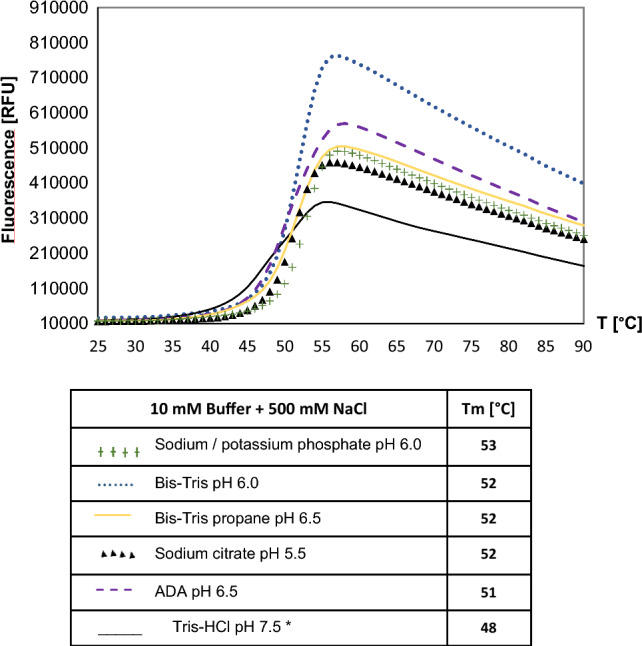
Thermofluor results of DrLigA: non normalized graphs and melting temperatures of the most stabilizing buffer conditions. *Reference buffer representing buffer 1

We performed thermal shift assays to identify optimum buffers for protein stability to increase the protein crystallization probability (Ericsson et al. 2006). Results of the melting curves of DrLigA showed that the protein was stable over a pH range from 4.5 to 10 (Supplementary Fig. S2a, d). We assessed the curves shape and also the curves height, as an increase in RFU implies an increase in the protein solubility. The melting temperature (T_m_) for each buffer condition was also determined, because an increase in T_m_ implies an increase in the protein thermostability. Based on the melting curves and T_m_, the most stabilizing buffers were considered as the sodium/potassium phosphate pH 6.0, Bis–Tris pH 6.0, Bis–Tris propane pH 6.5, and sodium citrate pH 5.5 (Fig. 2). These buffers produced 3 °C to 5 °C increase in T_m_ relative to the reference buffer representing buffer 1 (Fig. 2). Although phosphate buffers provided higher T_m_, they are known to form salt crystals which is problematic during crystallization experiments. Therefore, we decided to use Bis–Tris pH 6.0 in subsequent purification experiments.

Additionally, we compared the thermostability between DrLigA and DrLigA∆BRCT, to analyze the effect of BRCT deletion on protein’s thermostability. The thermofluor results indicated that DrLigA∆BRCT presents similar T_m_, as the full-length variant, and thus, no major effect was observed due to BRCT deletion (Supplementary Fig. S3). Moreover, the thermostability of DrLigA (in buffer 2) was assessed with different additives: 300 µM NAD^+^, 1 mM Mg^2+^, 1 mM Mn^2+^, or 1 mM Zn^2+^. We observed that curves and T_m_ values were similar for each additive, except for the condition with 1 mM Zn^2+^ (Supplementary Fig. S4). With 1 mM Zn^2+^, we detected an atypical curve with two T_m_ transitions, indicating a differential stabilization of DrLigA domains upon Zn^2+^ binding (Supplementary Fig. S4).

### Neutral pH and absence of salt are optimal conditions for nick-closure activity

Previous analysis of DNA ligation nick sealing activity of DrLigA has been demonstrated by gel-based activity assays with labelled DNA substrates. The results of Blasius et al. (2007) showed that ligation was optimal with 1 mM MnCl_2_ and pH 6.8, and that higher concentrations than 5 µM NAD^+^ had an inhibitory effect on the activity. The results of Le et al. (2008) showed that activity was better with 5 mM MnCl_2_ or MgCl_2_, although the enzyme preferred MnCl_2_. The activity was optimal at pH 7.0, and 10–20 mM of NaCl or KCl, and with 1.5 mM of NAD^+^.

**Fig. 3:**
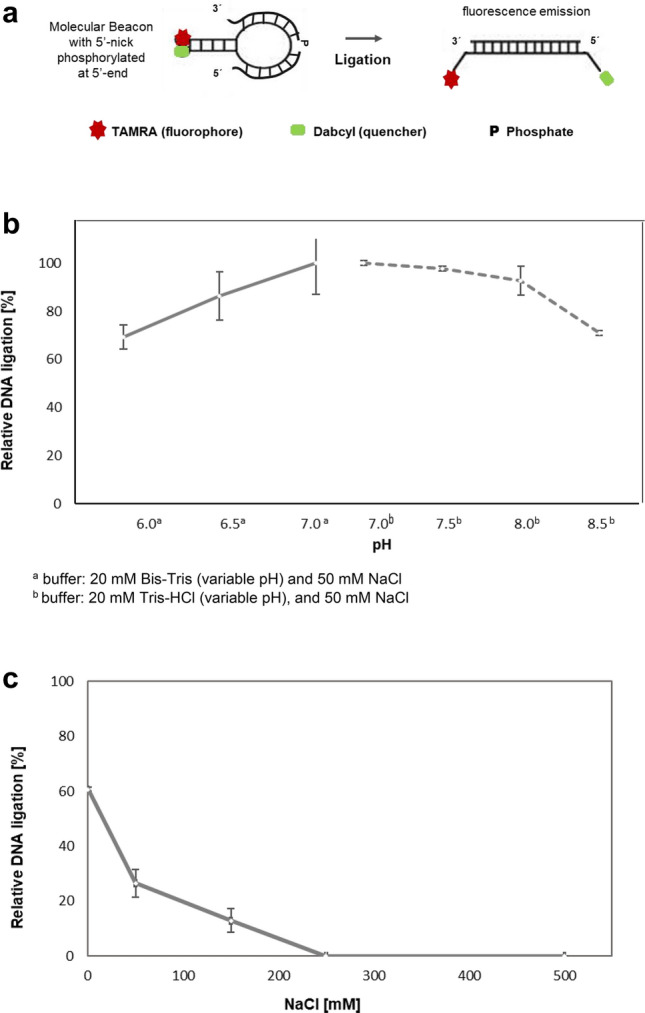
Molecular beacon-based activity assays with 330 nM of DrLigA: DNA ligation by nick closure. **a** Representation of the molecular beacon with a single 5′-nick which is phosphorylated at the 5′-end and contains the fluorophore–quencher pair TAMRA-Dabcyl. **b** and **c** The increase in fluorescence was measured as relative fluorescence units (RFU) over time, and the DNA ligation activity is represented as the initial velocity, which data were normalized to 100%. **b** pH effect: dotted line—buffer 20 mM Tris–HCl 50 mM NaCl, solid line—20 mM Bis–Tris 50 mM NaCl. **c** Salt concentration effect: experiment performed with 5 μM NAD^+^ in buffer 20 mM Bis–Tris pH 7.0, 50 mM NaCl. Experiments were performed in duplicate. Error bars represent 1 standard deviation from the mean (1 S.D.)

To clarify some of these results, the molecular beacon assay as described by (Tang et al. 2003) was used. When ligation reaction occurs, the nick is closed, and a longer DNA strand is complementary to the molecular beacon leading it to open, and subsequently, there is fluorescence emission (Fig. 3a). By comparing to the commonly used discontinuous assay via denatured gel electrophoresis and autoradiography, activity molecular beacon-based assay is faster, more sensitivity, more specific and allows real-time monitoring of the ligation activity (Tang et al. 2003). Using this molecular beacon assay, we have characterized DNA nick sealing of DrLigA at different pHs, salt, and NAD^+^ concentrations. Since some metals (e.g., Mn^2+^) affect fluorescence emission at higher concentrations, we were not able to assess the influence of metals using this approach. Thus, 1 mM Mn^2+^ was used for all assays, based on the previously determined optimum metal (Blasius et al. 2007).

All assays were carried out at 30 °C, the optimal temperature for growing *D. radiodurans*. We confirmed that DrLigA is active across a wide pH range (pH 6.0 to 8.5) and has optimal activity at pH 7.0 (Fig. 3b). To complement and validate these results, a gel-based activity assay was also performed which demonstrated the formation of a 40 nt DNA ligation product across the same pH range (Supplementary Fig. S5).

**Fig. 4:**
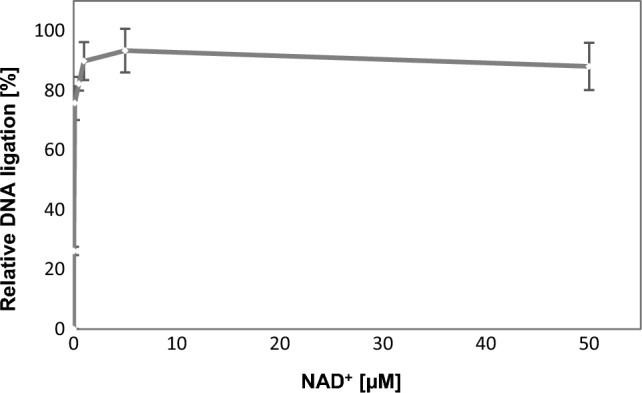
Molecular beacon-based activity assays with 170 nM of DrLigA: NAD^+^ concentration influence on nick closure. The increase in fluorescence was measured as relative fluorescence units (RFU) over time, and the DNA ligation activity is represented as the initial velocity, which data were normalized to 100%. Experiments were performed with 20 mM buffer Bis–Tris pH 6.5 and 50 mM of NaCl. Error bars represent 1 standard deviation from the mean (1 S.D.)

Using the molecular beacon-based assays, we also determined that the ligation activity is optimal in the absence of NaCl (Fig. 3c). We analyzed NAD^+^ influence (0–50 μM of NAD^+^) to understand whether sub- or super-stochiometric concentration of cofactor stimulated or inhibited the ligation reaction. The results demonstrated that an increasing concentration of NAD^+^ (≥ 0.1 μM) favored DNA nick sealing (Fig. 4).

Overall, our results agree with the previous findings confirming that DrLigA has optimal activity at pH 7.0 (Blasius et al. 2007; Le et al. 2008). However, we also observed that the protein is more thermostable at pH 6.0, and at pH 6.0, the ligation efficiency was reduced to about 14% in comparison to pH 7.0 (Fig. 3b). We also confirmed the activity preference for low ionic strength buffers. Moreover, no inhibitory effect was induced by addition of excess NAD^+^ within the concentration range tested.

### DrLigA∆BRCT presents lower binding affinity to dsDNA without losing DNA ligation activity

**Fig. 5:**
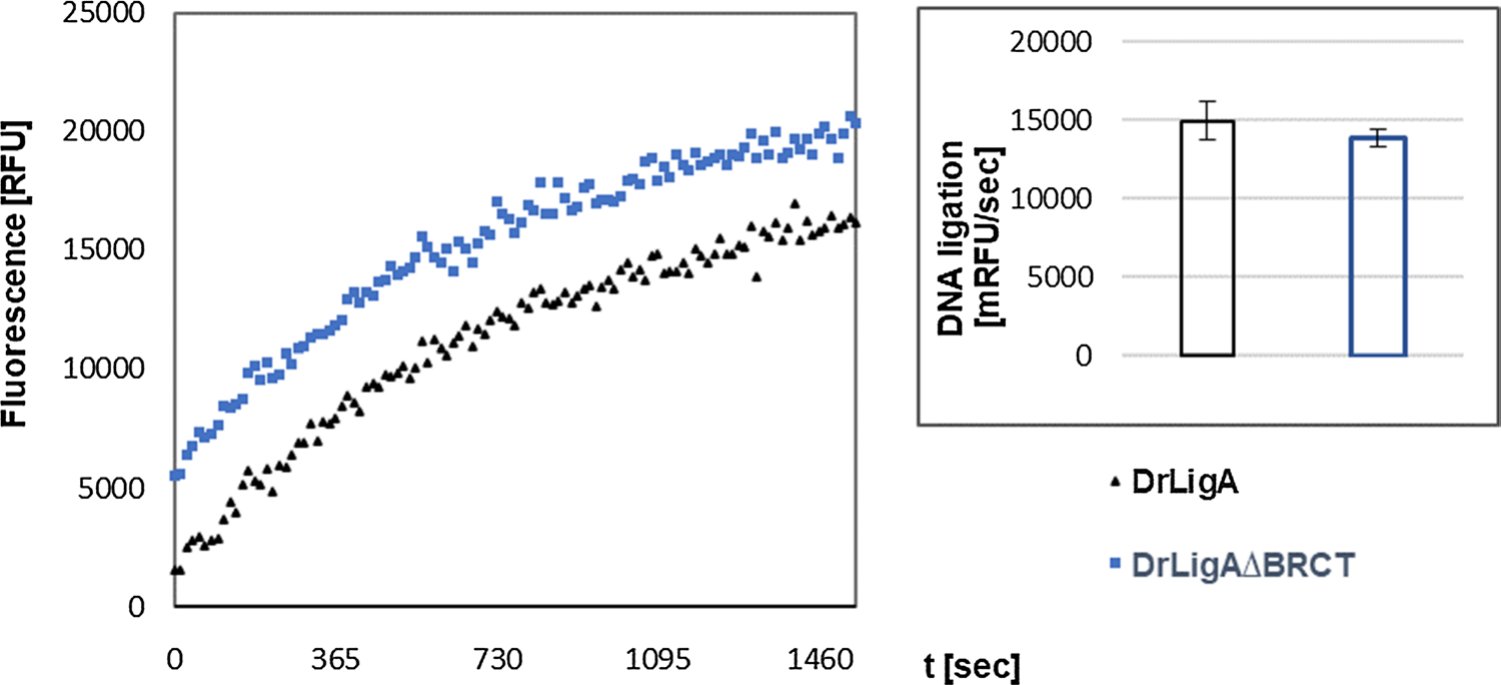
Molecular beacon-based activity assay with 66 nM of protein: DNA ligation by nick closure with DrLigA full-length versus truncated DrLigA∆BRCT. The increase in fluorescence was measured as relative fluorescence units (RFU) over time, and the ligation activity is represented as the initial velocity in mRFU per sec. Experiments performed at pH 7.0, with 1 mM Mn^2+^ and 5 µM NAD^+^. Curves represent the mean values (duplicates). Column graph error bars represent 1 standard deviation from the mean (1 S.D.)

After the characterization of DNA nick sealing by DrLigA, the activities of full-length and truncated proteins were compared under optimal reaction conditions (5 µM NAD^+^, and 1 mM Mn^2+^ in 20 mM Bis–Tris pH 7.0). DrLigA and DrLigA∆BRCT presented nearly the same efficiency to seal the 5′-nick of the molecular beacon. We compared the reaction at two different protein concentrations, 66 or 330 nM (Fig. 5, Supplementary Figure S6). Both protein variants show similar ligation rates; therefore, the deletion of the BRCT domain did not appear to affect DNA ligation. Our result is consistent with the previous studies of LigA from *E. coli* (EcLigA), where it was shown that the BRCT truncated version had significant activity, although less than the full-length protein (Wilkinson et al. 2005). They also showed that the BRCT domain was not required for ligation in vivo. An *E. coli* strain GR501, with a temperature-sensitive mutation to LigA, grew at a non-permissive temperature when the truncated EcLigA without BRCT was over-expressed. Additionally, Jeon et al. (2004) analyzed *Thermus filiformis* LigA (ThLigA), and the protein variant without BRCT domain presented nick-closure activity in vitro and in vivo, although the enzymatic efficiency was approximately 50% less than the wild-type protein. They also showed that the isolated fragment of BRCT of ThLigA presented DNA-binding activity but no ligation activity. Feng et al. (2004) reported that an absent or modified BRCT domain affected the ligation catalysis. They studied *Thermus* species and analyzed the effect of deletions or mutations in the BRCT domain. The BRCT domain deletion variant and the mutant G617I of ThLigA showed a lower ligation activity (~ 20% less than the wild type). However, this activity reduction was only detectable in reactions with excess of enzyme. Moreover, because of the lack of accumulation of an AMP–LigA intermediate, they suggested that these modifications on the BRCT of ThLigA affected the protein's activity at steps after adenylation. All these studies detected decreased DNA binding by truncated LigAs without the BRCT domain.

**Fig. 6:**
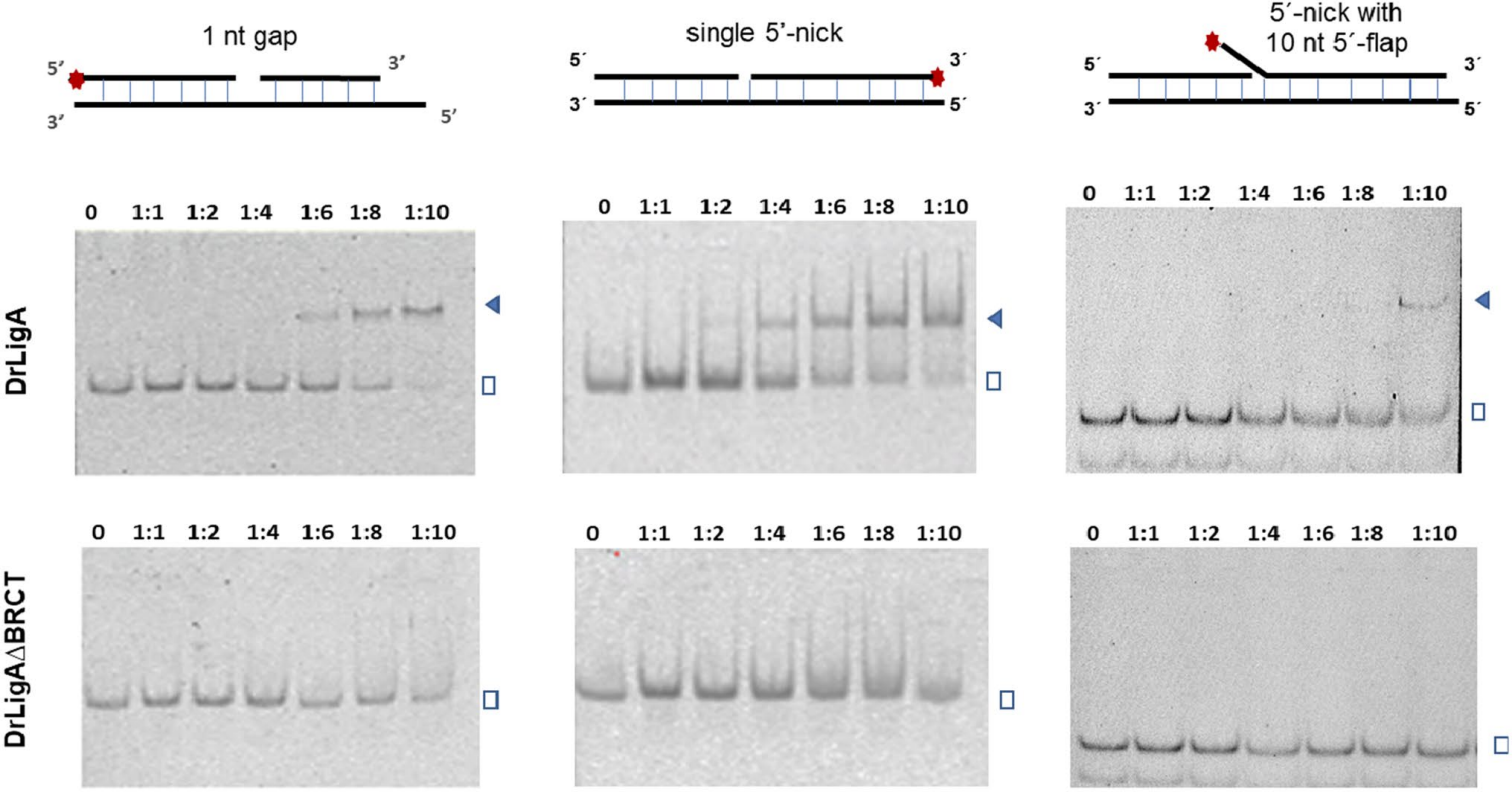
Gel-shift assays with 100 nM of FAM-labelled DNA substrates: DNA binding of DrligA full-length versus truncated DrLigA∆BRCT. The incubation of DNA substrates (with the 5′-end of the internal nick/gap not phosphorylated) and proteins was performed for 15 min at room temperature and an increasing concentration of protein was added. 0, 1:1, 1:2, 1:4, 1:6, 1:8, and 1:10 represent the molar ratio of [DNA]: [protein]. The blue triangles and the open squares indicate the DNA–protein complex and free DNA, respectively

We assessed the binding of the full-length DrLigA and DrLigA∆BRCT to dsDNA using gel mobility shift assays. Different 40 nt oligos that were not phosphorylated (to ensure no ligation activity) were used: (i) 1 nt gapped dsDNA; (ii) a single nicked dsDNA; and (iii) a single nicked dsDNA with a 10 nt 5′-flap (Fig. 6). We observed that the DrLigA–DNA complex was formed with increasing concentrations of protein. The binding was tight to nicked dsDNA and gapped dsDNA, when the protein concentration was ten fold higher than the DNA concentration. However, at this concentration, the affinity of DrLigA to dsDNA with a 5′-flap was weak (Fig. 6). The 5′-flap probably interfered in the protein interaction with the DNA, because the flap sterically hindered the binding of subdomain 1b to the nick site. Based on the higher affinity of the protein to dsDNA with a nick and 1 nt gap, DrLigA appears to recognize unphosphorylated DNA nicks and gaps. Nick or target site recognition is crucial for the DrLigA action.

On the contrary, no DrLigA∆BRCT–DNA complex band was detected for all oligos used within the range of the protein concentrations analyzed (Fig. 6). It was clear that the deletion of the BRCT domain resulted in substantial loss of enzyme affinity to dsDNA in comparison to DrLigA. Our results showing the reduced affinity of DrLigA∆BRCT to dsDNA support the generally accepted hypothesis that the BRCT domain is important for the DNA binding process or nick recognition.

Conserved motifs and functional sites among prokaryotic and eukaryotic BRCT domains have been already identified. According to (Callebaut and Mornon 1997), BRCT contains five hydrophobic motifs, and they include phosphoserine/phosphothreonine-binding modules that were structurally analyzed in human proteins in complex with phosphorylated peptides (Clapperton et al. 2004; Williams et al. 2004). Besides protein interactions, eukaryote BRCT domains have been implicated in diverse functions, DNA binding, phosphorylation-independent protein interactions, poly(ADP-ribose) binding, and in regulatory mechanisms (Leung and Glover 2011). In prokaryotes, the BRCT phosphate-binding pocket is associated with the binding of phosphates at the DNA nick or DNA ends. Indeed, the functional BRCT phylogenetic tree reconstruction by Sheng et al. (2011) indicated that in eukaryotes, this phosphate-binding function of BRCT diverged and is conserved in prokaryotes. Sheng et al. (2011) proposed that the bacterial BRCT is the ancestor of eukaryote BRCT domains, and this functional diversification in eukaryotes was driven by the evolution of their DNA damage response mechanisms.

### DrLigA∆BRCT crystallization and X-ray data

**Fig. 7:**
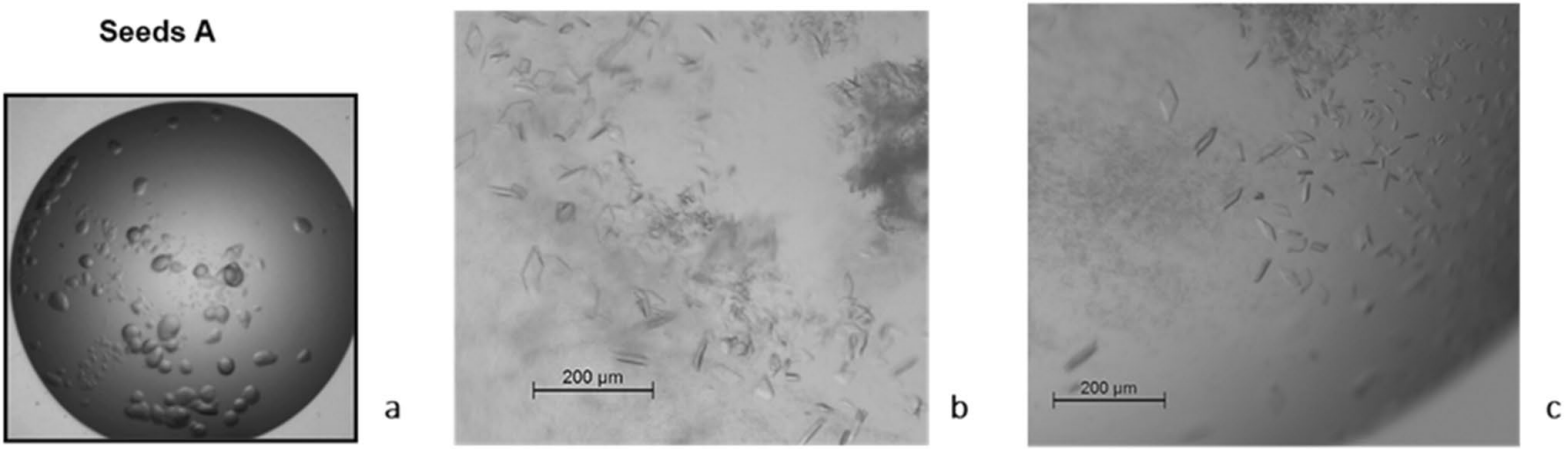
DrLigA and DrLigA∆BRCT crystallization results: **a** DrLigA spherulites obtained in solution A (1 M sodium acetate trihydrate, 0.1 M sodium HEPES pH 7.5, 0.05 M cadmium sulfate 8/3 hydrate) by sitting drop vapor diffusion. They were used for seeding (seeds A). **b** and **c** Crystals of DrLigA∆BRCT obtained by microseeding with seeds A. **b** DrLigA∆BRCT crystals grew in solution B (20% w/v PEG 8000, 0.2 M magnesium acetate tetrahydrate, and 0.1 M sodium cacodylate pH 6.5). **c** DrLigA∆BRCT crystals obtained in solution B with 0.01 M trimethylamine hydrochloride as additive

Crystallization experiments of full-length DrLigA resulted in spherulites which were used to make seed stocks A (Fig. 7a). Microseed matrix screening experiments with seeds A improved the crystallization efficiency of DrLigA, although most of the crystals were clusters of small needles which failed to produce high-quality diffraction data (Supplementary Fig. S7).

To date, two full-length bacterial ligases structures are known: ThLigA (Lee et al. 2000) and EcLigA (Nandakumar et al. 2007; Unciuleac et al. 2017). In these structures, no or poor electron density was observed for the BRCT C-terminal domain, indicating that it is a disordered or a very dynamic domain. However, it was still identified on ThLigA structure that the domain displays four parallel β-sheet strands flanked by three helices (Lee et al. 2000). Due to the potential high mobility of BRCT domain, our next approach was to remove this domain in an attempt to obtain crystals. With the deletion of BRCT domain both crystallization efficiency and crystal quality improved (Fig. 7b, c, Supplementary Table S4). Data were collected from crystals grown in the presence of the additive 0.01 M trimethylamine hydrochloride (Fig. 7c) which diffracted to a resolution of 3.4 Å. 530 images were collected, data were processed, and after an anisotropic resolution cut-off, MR was implemented as the phasing method. MR search with fragments/ensembles from the predicted AlphaFold2 structure gave a clear and single solution. The structure was built, corrected, and refined with final R-work/R-free values of 0.215/0.275. Due to poor electron density at the beginning of the N-terminus and end of C-terminus, the structural model only contains residues 81–593. Non-protein electron density was modeled as two Mn^2+^ in the catalytic pocket, one Zn^2+^ in the Zn-finger domain, and three extra Zn^2+^ cations at the protein surface. Although these extra cations have been modeled as Zn^2+^, their chemical identity is yet to be confirmed. Complete information about X-ray data collection, processing, and structural refinement is shown in Table 3. The final refined protein coordinates and experimental structure factors were submitted to the Protein Data Bank (Burley et al. 2019) with accession code 8AK4.

### The structure of DrLigA∆BRCT

**Fig. 8:**
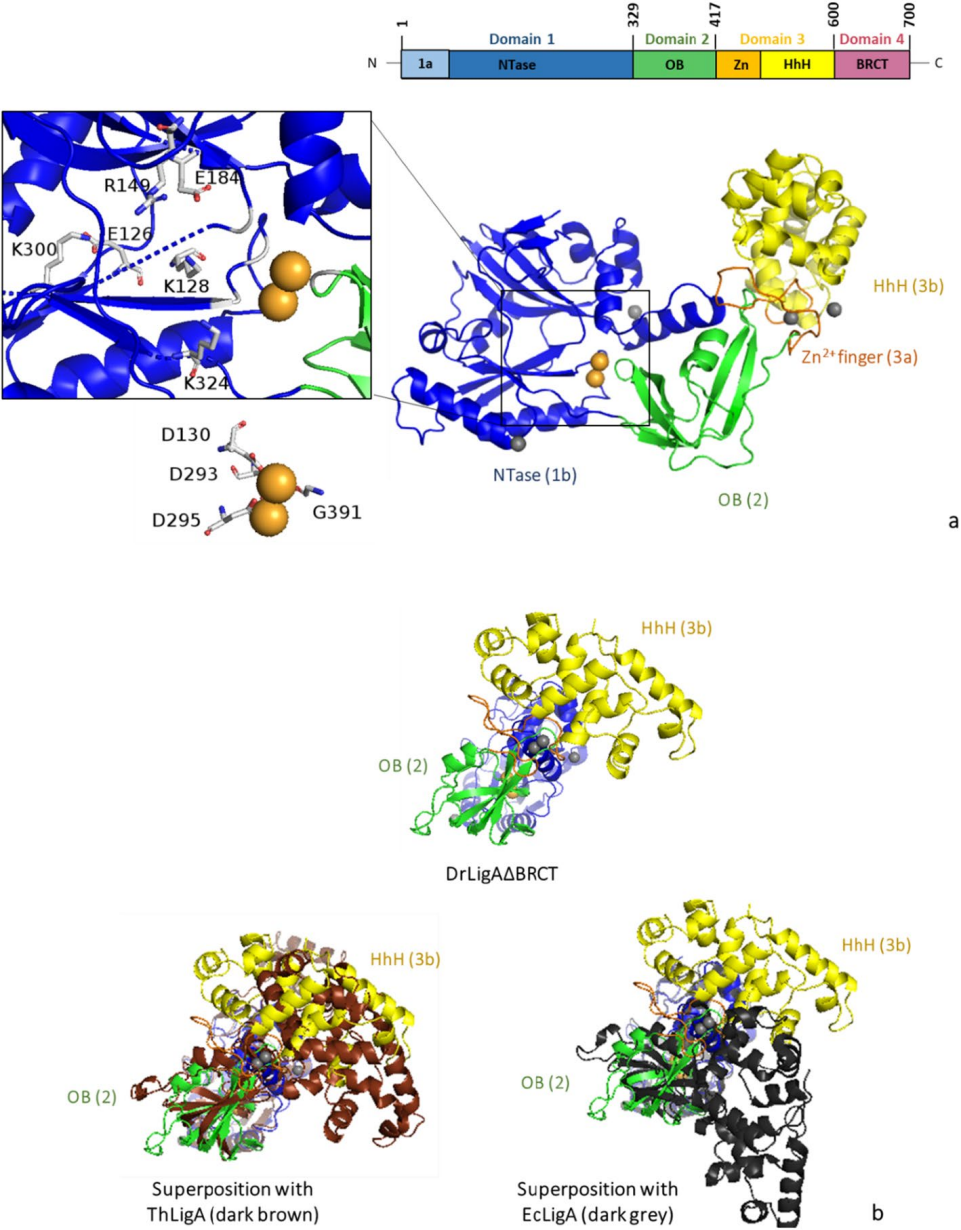
Structure of DrLigA∆BRCT: **a** modular organization of DrLigA and overall structure of DrLigA∆BRCT with NTase subdomain 1b (blue), OB-fold domain 2 (green), zinc finger motif 3a (orange), and HhH subdomain 3b (yellow) identified. On the left, amplified view of the two-metal center and catalytic site. K128 from KXDG motif, residues E126, R149, E184, K300, and K324 (predicted to interact with NAD^+^) (top view), and the residues D130, D293, D295, and G391 (predicted to interact with Mn^2+^) (bottom view) are labelled. Mn^2+^ cations are represented as light orange spheres, and Zn^2+^ cations as light gray spheres. **b** Representation of superimposed DrLigA∆BRCT (alignment of subdomain 1b) with ThLigA structure (PDB ID: 1dgs, dark brown) and with EcLigA structure (PDB ID: 5tt5, dark gray). *NTase* nucleotidyl transferase, *OB* oligonucleotide-binding, *Zn* zinc finger, *HhH* helix–hairpin–helix, *BRCT* breast cancer type 1 C-terminus

We obtained crystals of DrLigA∆BRCT comprising subdomain 1a, subdomain 1b, domain 2, subdomain 3a, and subdomain 3b. However, no electron density was observed for the N-terminal subdomain 1a, and residues 1–78 are not represented in our structure. Our DrLigA∆BRCT structure displays a modular architecture, consisting of: (i) an NTase subdomain 1b (residues 79–328) that comprises antiparallel β-sheets flanked by α-helices; (ii) an OB-fold domain 2 (residues 329–416) that folds as an antiparallel β-barrel; (iii) subdomain 3a is a 4-Cys (C418, C421, C436, C441) zinc finger with one bound Zn^2+^ ion, and iv) HhH subdomain 3b (residues 442–594) forms a 4 helix–hairpin–helix motif (Fig. 8a).

Due to the lack of electron density for the N-terminal subdomain 1a, it was not possible to assess whether subdomain 1a buries the adenylation pocket (closed conformation) or if the adenylation active site is exposed (open conformation). Subdomain 1a has to turn by almost 180° to shift from open to a closed conformation (Gajiwala and Pinko 2004). These conformational changes are important for NAD^+^ recognition and protein adenylation. Because we purified DrLigA∆BRCT with subdomain 1a, but it was undetected, we infer that subdomain 1a was adopting different conformations in the crystal structure. We could therefore not determine whether NAD^+^ or NMM is bound to this subdomain. Neither NAD^+^ nor AMP groups were found in the catalytic pocket bound to the conserved K128. Thus, our DrLigA∆BRCT structure is non-adenylated. However, we identified K128, and the residues which are predicted to interact with NAD^+^ (E126, R149, E184, K300, and K324) (Fig. 8a). Interestingly, we identified an unexpected two-metal center that stabilized the empty catalytic site. It is considered that in adenylation, LigAs follow one-metal mechanism (Unciuleac et al. 2017). In our structure, the two putative Mn^2+^ cations placed in the catalytic site are predicted to interact with residues D293, D295, G391 and also residue D130 from the conserved motif KXDG (Fig. 8a). However, due to the low structure resolution, we did not further explore the coordination details of the two-metal center (Fig. 8a). The two-metal center may be result of the addition of MnSO_4_ to the protein solution prior to the crystallization experiments, or be of importance for the pre-adenylated state.

By aligning the NTase subdomain 1b with the corresponding subdomain of EcLigA-(K115M) (PDB ID: 5tt5) and ThLigA (PDB ID: 1dgs) structure, we detected a rotation between subdomain 1b and the domain 2, this rotation was more significant in comparison to EcLigA-(K115M) (Fig. 8b). The EcLigA-(K115M) structure also represents a non-adenylated enzyme but contains NAD^+^ and Mg^2+^ in the catalytic pocket (Unciuleac et al. 2017). The ThLigA structure represents an adenylated LigA covalently bound to AMP (Lee et al. 2000). Considering different reaction stages and conformations of LigA, we could infer that our structure of DrLigA∆BRCT represents a pre-adenylation stage of the enzyme. Then, EcLigA-(K115M)·NAD^+^·Mg^2+^ is in a pentavalent transition state of adenylation, with subdomain 1a in a closed conformation and the C-terminal domain in an extended conformation. Finally, the ThLigA-AMP structure (devoid of metals) represents an adenylated intermediate state, in an open conformation with the catalytic site exposed for DNA binding. Upon DNA binding, it is known that LigAs adopt a closed clamp form. EcLigA–dsDNA complex showed that upon DNA binding, a nearly 180° rotation of the domain 2 occurred for the protein–DNA clamp formation (Nandakumar et al. 2007).

As a classical NAD^+^-dependent ligase, our structure of DrLigA∆BRCT presents common structural features of LigA proteins. The high degree of homology between these enzyme sequences (Supplementary Fig. S8) not only indicates structural similarities but also suggests identical catalytic mechanisms. The determinations of different LigAs structures have been unveiling these mechanisms. Nonetheless, nick recognition process remains unclear. The determination of different LigA–DNA intermediates will potentially provide a comprehensive view of the structural basis of DNA nick recognition. Thus, the determination of DrLigA in complex with different modified nick substrates is a future aim, not only for deepen our understanding about nick recognition mechanisms but also to increase the probability to solve BRCT domain structure, this domain may be stabilized by interacting with DNA.

## Concluding remarks

We have characterized recombinant DrLigA and truncated DrLigAΔBRCT biochemically and structurally. Activity analysis of the full-length protein with molecular beacon assays confirmed that nick sealing is optimal at neutral pH and in the absence of salt. Moreover, the ligase activity of DrLigAΔBRCT was preserved, but its affinity to dsDNA decreased in comparison to full-length DrLigA, and reinforce the assumption that the C-terminal BRCT domain 4 is involved in DNA binding.

Since no structure of BRCT of LigAs has been determined in complex with DNA, it is unknown how the domain binds to DNA. However, other full-length LigA structures have given some insights into DNA binding via other domains. Based on the ThLigA structure, two putative DNA-binding sites were predicted: (i) the ‘catalytic DNA-binding site’, which comprises the adenylation site and is placed in the interface between NTase subdomain 1b and domain 2; and (ii) the ‘non-catalytic DNA-binding site’ that includes the HhH motifs of domain 3b. Both DNA-binding sites were indicated to interact independently with DNA (Lee et al. 2000). Additional evidence about LigA–DNA interactions were revealed based on the only known structure of LigA in complex with dsDNA (Nandakumar et al. 2007). The EcLigA–DNA complex structure showed that subdomain 1b binds at the DNA nick and its flanking sites. The OB-fold domain 2 binds the template strand of the DNA nick. Therefore, the NTase subdomain 1b and OB-fold domain 2 do indeed contain the ‘catalytic DNA-binding site’. The zinc finger domain 3a bridges the OB domain 2 to the HhH domain 3b, and the HhH motifs interact with the two strands of the DNA minor groove. The EcLigA–DNA complex structure shows that the enzyme encircles the DNA forming a clamp. The LigA clamp closes via contacts between its NTase subdomain 1b and HhH domain 3b (Nandakumar et al. 2007).

**Fig. 9:**
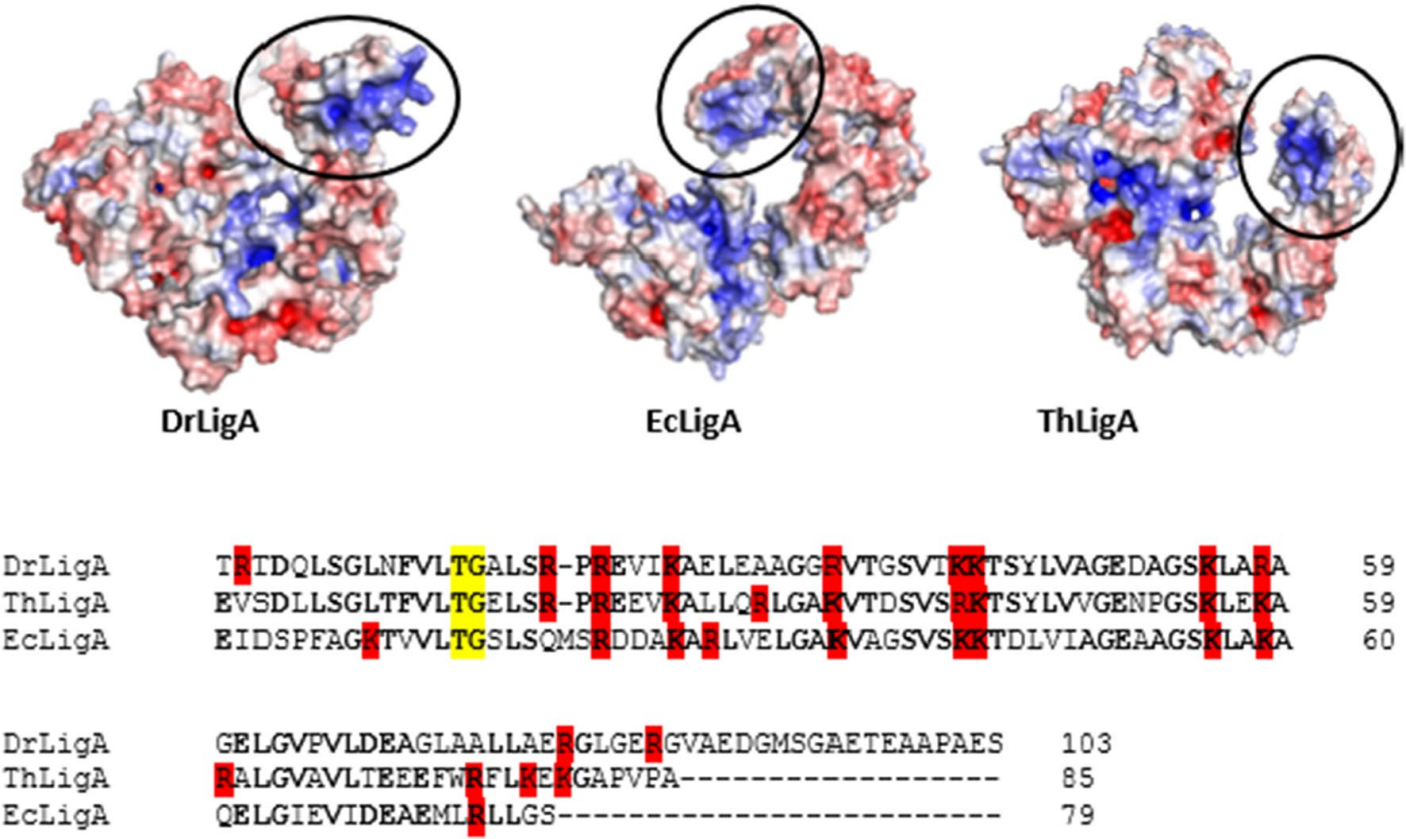
Electrostatic surface (blue positive charges, red negative charges) from bacterial DNA ligases A structures predicted by AlphaFold2—DrLigA (*D. radiodurans,* UniProtKB: Q9RSQ5), EcLigA (*E. coli*, UniProtKB: P15042), ThLigA (*T. filiformis*, UniProtKB: Q9ZHI0). Black ellipse identifies the BRCT domain. Multiple sequence alignment from Clustal Omega (https://www.ebi.ac.uk/Tools/msa/clustalo/), alignment of BRCT domain from DrLigA, ThLigA, and EcLigA. Positively charged residues Arg and Lys are highlighted in red. Conserved residues from the phosphate-binding pocket are colored yellow

To analyze the C-terminal BRCT domain, the BRCT structures of EcLigA, ThLigA, and DrLigA predicted by AlphaFold2 were compared (Fig. 9). Based on these AlphaFold structures, the BRCT domain is predicted to adopt different orientations/positions. Additionally, it is predicted that these BRCT domains encompass a positively charged surface, containing arginines and lysines, which are conserved between EcLigA, ThLigA, and DrLigA sequences (Fig. 9). This surface may interact with DNA and may be part of the putative ‘non-catalytic DNA-binding site’ alongside with HhH domain 3b; however, this needs further investigation. Taken together, it is likely the BRCT domain possesses a significant role in DNA binding in LigA-type DNA ligases.

### Supplementary Information

Below is the link to the electronic supplementary material.

Supplementary file1 (DOCX 2835 KB)

## Data Availability

The final refined protein coordinates and experimental structure factors were submitted to the Protein Data Bank (Burley et al. 2019) with accession code 8AK4.

## Abbreviations

AMP: Adenosine monophosphate
ATP: Adenosine triphosphate
BSA: Bovine serum albumin
DNA: Deoxyribonucleic acid
DNase: Deoxyribonuclease
dNTPs: Mix of deoxynucleotide triphosphates
dsDNA: Double-stranded DNA
His_6_-tag: Hexa histidine-tag
NAD^+^: Nicotinamide adenine dinucleotide
NMN: Nicotinamide mononucleotide
nt: Nucleotides
PAGE: Polyacrylamide gel electrophoresis
TBE: Tris–Borate–EDTA buffer

## References

[CR1] Blasius M, Buob R, Shevelev IV, Hubscher U (2007). Enzymes involved in DNA ligation and end-healing in the radioresistant bacterium *Deinococcus radiodurans*. BMC Mol Biol.

[CR2] Burley SK, Berman HM, Bhikadiya C (2019). Protein data bank: the single global archive for 3D macromolecular structure data. Nucleic Acids Res.

[CR3] Callebaut I, Mornon JP (1997). From BRCA1 to RAP1: a widespread BRCT module closely associated with DNA repair. FEBS Lett.

[CR4] Clapperton JA, Manke IA, Lowery DM (2004). Structure and mechanism of BRCA1 BRCT domain recognition of phosphorylated BACH1 with implications for cancer. Nat Struct Mol Biol.

[CR5] Cowtan K (2006). The Buccaneer software for automated model building. 1. Tracing protein chains. Acta Crystallogr D Biol Crystallogr.

[CR6] Bond PS, Cowtan K (2022). ModelCraft: an advanced automated model-building pipeline using Buccaneer. Acta Crystallogr D Struct Biol.

[CR7] D’Arcy A, Villard F, Marsh M (2007). An automated microseed matrix-screening method for protein crystallization. Acta Crystallogr D Biol Crystallogr.

[CR8] Emsley P, Lohkamp B, Scott WG, Cowtan K (2010). Features and development of coot. Acta Crystallogr D Biol Crystallogr.

[CR9] Ericsson UB, Hallberg BM, DeTitta GT (2006). Thermofluor-based high-throughput stability optimization of proteins for structural studies. Anal Biochem.

[CR10] Feng H, Parker JM, Lu J, Cao W (2004). Effects of deletion and site-directed mutations on ligation steps of NAD^+^-dependent DNA ligase: a biochemical analysis of BRCA1 C-terminal domain. Biochemistry.

[CR11] Fernandes A, Piotrowski Y, Williamson A (2021). Studies of multifunctional DNA polymerase I from the extremely radiation resistant *Deinococcus radiodurans*: recombinant expression, purification and characterization of the full-length protein and its large fragment. Protein Exp Purif.

[CR12] Gajiwala KS, Pinko C (2004). Structural rearrangement accompanying NAD^+^ synthesis within a bacterial DNA ligase crystal. Structure.

[CR13] Jeon HJ, Shin HJ, Choi JJ (2004). Mutational analyses of the thermostable NAD^+^-dependent DNA ligase from *Thermus filiformis*. FEMS Microbiol Lett.

[CR14] Juanhuix J, Gil-Ortiz F, Cuní G (2014). Developments in optics and performance at BL13-XALOC, the macromolecular crystallography beamline at the alba synchrotron. J Synchrotron Radiat.

[CR15] Jumper J, Evans R, Pritzel A (2021). Highly accurate protein structure prediction with AlphaFold. Nature.

[CR16] Jurrus E, Engel D, Star K (2018). Improvements to the APBS biomolecular solvation software suite. Protein Sci.

[CR17] Kabsch W (2010). XDS. Acta Crystallogr D Biol Crystallogr.

[CR18] Kota S, Kamble VA, Rajpurohit YS, Misra HS (2010). ATP-type DNA ligase requires other proteins for its activity in vitro and its operon components for radiation resistance in *Deinococcus radiodurans* in vivo. Biochem Cell Biol.

[CR19] Kovalevskiy O, Nicholls RA, Long F (2018). Overview of refinement procedures within REFMAC 5: utilizing data from different sources. Acta Crystallogr D Struct Biol.

[CR20] Le D, Hua X, Huang L (2008). Biochemical characterization of two DNA ligases from *Deinococcus radiodurans*. Protein Pept Lett.

[CR21] Lee JY, Chang C, Song HK (2000). Crystal structure of NAD+-dependent DNA ligase: modular architecture and functional implications. EMBO J.

[CR22] Lehman IR (1974). DNA ligase: structure, mechanism, and function. Science (1979).

[CR23] Leung CCY, Glover JNM (2011). BRCT domains: easy as one, two, three. Cell Cycle.

[CR24] Liebschner D, Afonine PV, Baker ML (2019). Macromolecular structure determination using X-rays, neutrons and electrons: recent developments in Phenix. Acta Crystallogr D Struct Biol.

[CR25] Liu Y, Zhou J, Omelchenko MV (2003). Transcriptome dynamics of *Deinococcus radiodurans* recovering from ionizing radiation. Proc Natl Acad Sci USA.

[CR26] McCoy AJ, Grosse-Kunstleve RW, Adams PD (2007). Phaser crystallographic software. J Appl Crystallogr.

[CR27] Nandakumar J, Nair PA, Shuman S (2007). Last stop on the road to repair: structure of *E. coli* DNA ligase bound to nicked DNA-adenylate. Mol Cell.

[CR28] Pascal JM (2008). DNA and RNA ligases: structural variations and shared mechanisms. Curr Opin Struct Biol.

[CR29] Pergolizzi G, Wagner GK, Bowater RP (2016). Biochemical and structural characterization of DNA ligases from bacteria and archaea. Biosci Rep.

[CR30] Petit MA, Ehrlich SD (2000). The NAD^+^-dependent ligase encoded by yerG is an essential gene of *Bacillus subtilis*. Nucleic Acids Res.

[CR31] Sheng ZZ, Zhao YQ, Huang JF (2011). Functional evolution of BRCT domains from binding DNA to protein. Evol Bioinf.

[CR32] Singleton MR, Håkansson K, Timson DJ, Wigley DB (1999). Structure of the adenylation domain of an NAD^+^-dependent DNA ligase. Structure.

[CR33] Srivastava SK, Tripathi RP, Ramachandran R (2005). NAD^+^-dependent DNA ligase (Rv3014c) from *Mycobacterium tuberculosis*: crystal structure of the adenylation domain and identification of novel inhibitors. J Biol Chem.

[CR34] Tang Z, Wang K, Tan W, Li J, Liu L, Guo Q, Meng X, Ma C, Huang S (2003) Real-time monitoring of nucleic acid ligation in homogenous solutions using molecular beacons. Nucleic Acids Res 31:148e–1148. 10.1093/nar/gng146 10.1093/nar/gng146 PMC290283 14627838

[CR35] Tickle IJ, Flensburg C, Keller P (2018). STARANISO.

[CR36] Timson DJ, Singleton MR, Wigley DB (2000). DNA ligases in the repair and replication of DNA. Mutat Res.

[CR37] Tomkinson AE, Vijayakumar S, Pascal JM, Ellenberger T (2006). DNA ligases: structure, reaction mechanism, and function. Chem Rev.

[CR38] Unciuleac M-C, Goldgur Y, Shuman S (2017). Two-metal versus one-metal mechanisms of lysine adenylylation by ATP-dependent and NAD^+^-dependent polynucleotide ligases. Proc Natl Acad Sci.

[CR39] Vonrhein C, Flensburg C, Keller P (2011). Data processing and analysis with the autoPROC toolbox. Acta Crystallogr D Biol Crystallogr.

[CR40] White O, Eisen JA, Heidelberg JF (1999). Genome sequence of the radioresistant bacterium *Deinococcus radiodurans* R1. Science (1979).

[CR41] Wilkinson A, Day J, Bowater R (2001). Bacterial DNA ligases. Mol Microbiol.

[CR42] Wilkinson A, Smith A, Bullard D (2005). Analysis of ligation and DNA binding by *Escherichia coli* DNA ligase (LigA). Biochim Biophys Acta.

[CR43] Williams RS, Lee MS, Hau DD, Glover JNM (2004). Structural basis of phosphopeptide recognition by the BRCT domain of BRCA1. Nat Struct Mol Biol.

[CR44] Williams CJ, Headd JJ, Moriarty NW (2018). MolProbity: more and better reference data for improved all-atom structure validation. Protein Sci.

[CR45] Williamson A, Leiros HKS (2020). Structural insight into DNA joining: from conserved mechanisms to diverse scaffolds. Nucleic Acids Res.

[CR46] Winn MD, Ballard CC, Cowtan KD (2011). Overview of the CCP4 suite and current developments. Acta Crystallogr D Biol Crystallogr.

