# Supplementary material for: Structure/function studies of the NAD+-dependent DNA ligase from the poly-extremophile Deinococcus radiodurans reveal importance of the BRCT domain for DNA binding
Source: Extremophiles. 2023 Sep 15;27(3):26. doi: 10.1007/s00792-023-01309-z (PMC10504179; doi:10.1007/s00792-023-01309-z)
Supplement: Supplementary file 1 — Supplementary file1 (DOCX 2835 KB) [file 792_2023_1309_MOESM1_ESM.docx]

**Supplementary information**

Structure/function studies of the NAD^+^-dependent DNA ligase from the poly-extremophile *Deinococcus radiodurans* reveal importance of the BRCT domain for DNA binding

**Supplementary Table S1.** Cloning of DrLigA and DrLigAΔBRCT: List of primers.

| **Name** | **Sequence 5’ -> 3’** |
| --- | --- |
| FPDrLigA | CATCACCATCACCATCACGAAAACCTGTATTTCCAGGGAGCAGTGCGTTACCCTGGGCGCATGA |
| RPDrLigA | GGGGACCACTTTGTACAAGAAAGCTGGGTCTCAGCTTTCAGCGGGGGCCGCC |
| FDRalle | GGGGACAAGTTTGTACAAAAAAGCAGGCTTCGAAGGAGATAGAACCATGCATCACCATCACCATCAC |
| RPbrct | GGGGACCACTTTGTACAAGAAAGCTGGGTCTCAGGTGCGGGTCACTTCCTCGG |

**SupplementaryTable S2.** Description of the Thermofluor buffer screening conditions

**Supplementary Table S3.** List of commercial high-throughput protein crystallization screens used

| - - 1. Structure Screen 1 & 2 (Molecular Dimensions) |
| --- |
| - - 1. JCSG^+^ Screen (Molecular Dimensions) |
| - - 1. Morpheus (Molecular Dimensions) |
| - - 1. Macrosool (Molecular Dimensions) |
| - - 1. Stura FootPrint (Molecular Dimensions) |
| - - 1. SG1 ShotGun (Molecular Dimensions) |
| - - 1. PGA (Molecular Dimensions) |
| - - 1. BCS (Molecular Dimensions) |
| - - 1. JBScreen Nuc-Pro (Jena Bioscience) |

**Supplementary Table S4.** Results overview for microseed matrix screening: DrLigA versus DrLigA∆BRCT

| **Crystals** | **No. hits** | |
| --- | --- | --- |
|  | **DrLigA** | **DrLigA∆BRCT** |
| **JCSG+ Screen** | | |
| Spherulites | 1 | 9 |
| Cluster of needles/sea urchins | 6 | 9 |
| Plates | 0 | 5 |
| ‘3D crystals’ | 1 | 0 |
| **Structure Screen 1 & 2** | | |
| Spherulites | 2 | 6 |
| Cluster of needles/sea urchins | 9 | 11 |
| Plates | 0 | 6 |
| ‘3D crystals’ | 0 | 1 |

**
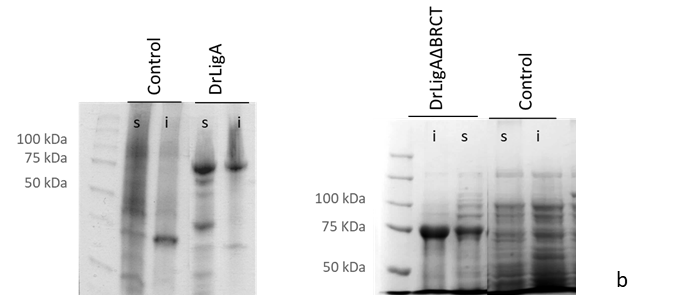
 a**

**
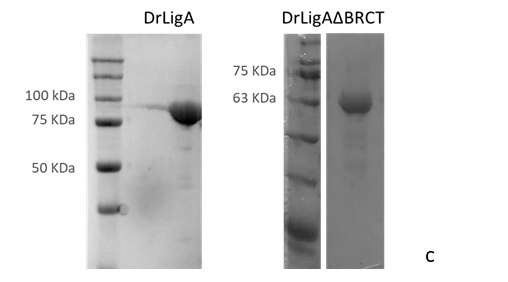
 b**

**Supplementary Figure S1.** Protein expression and purification: recombinant DrLigA and DrLigAΔBRCT. a) Protein expression in *E. coli* BL21* pRARE2, expression induced with 0.5 mM IPTG and performed for 3h at 37 °C. DrLigA and DrLigAΔBRCT overexpression in soluble (s) and insoluble fraction (i). Control - non induced cells. b) Analysis on SDS-PAGE gel of purified DrLigA (75.6 kDa) and DrLigAΔBRCT (65.5 kDa).

**
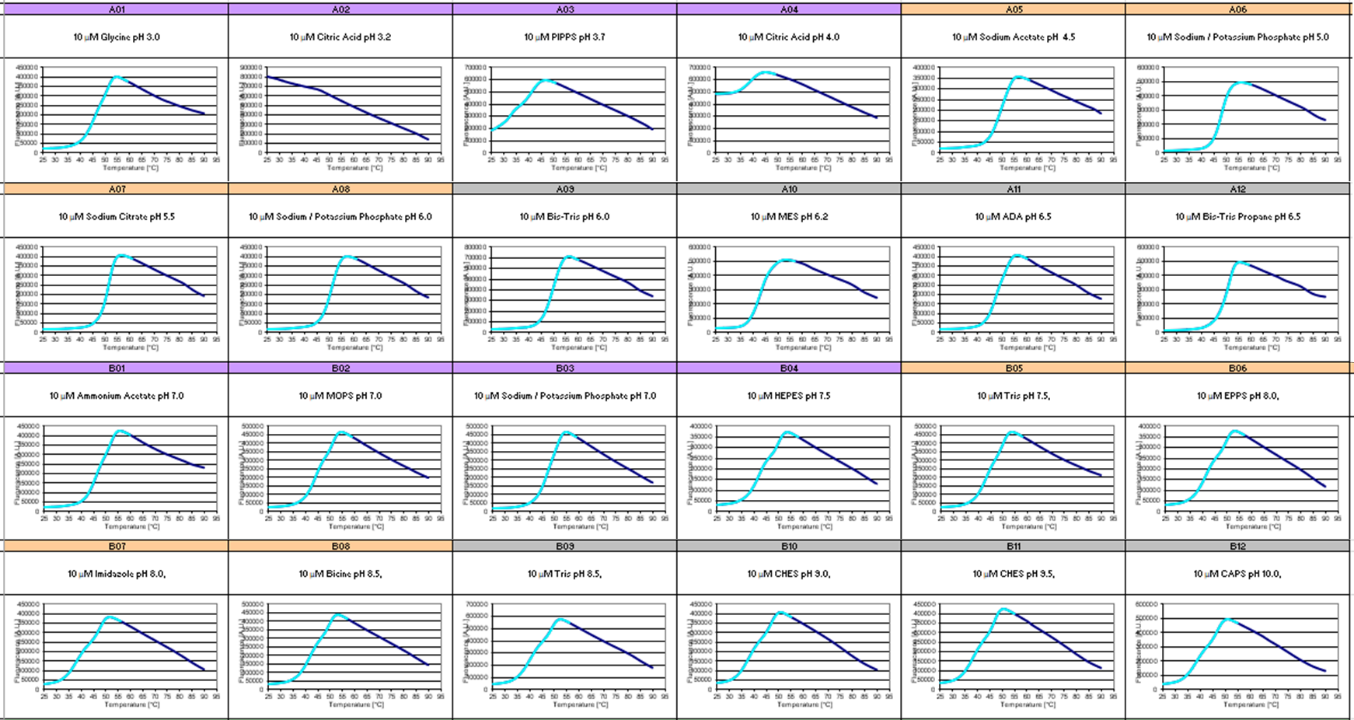
Supplementary Figure S2.** Thermofluor buffer screening with DrLigA: results overview (96-well plate): S2 a – curves from well A1 to B12


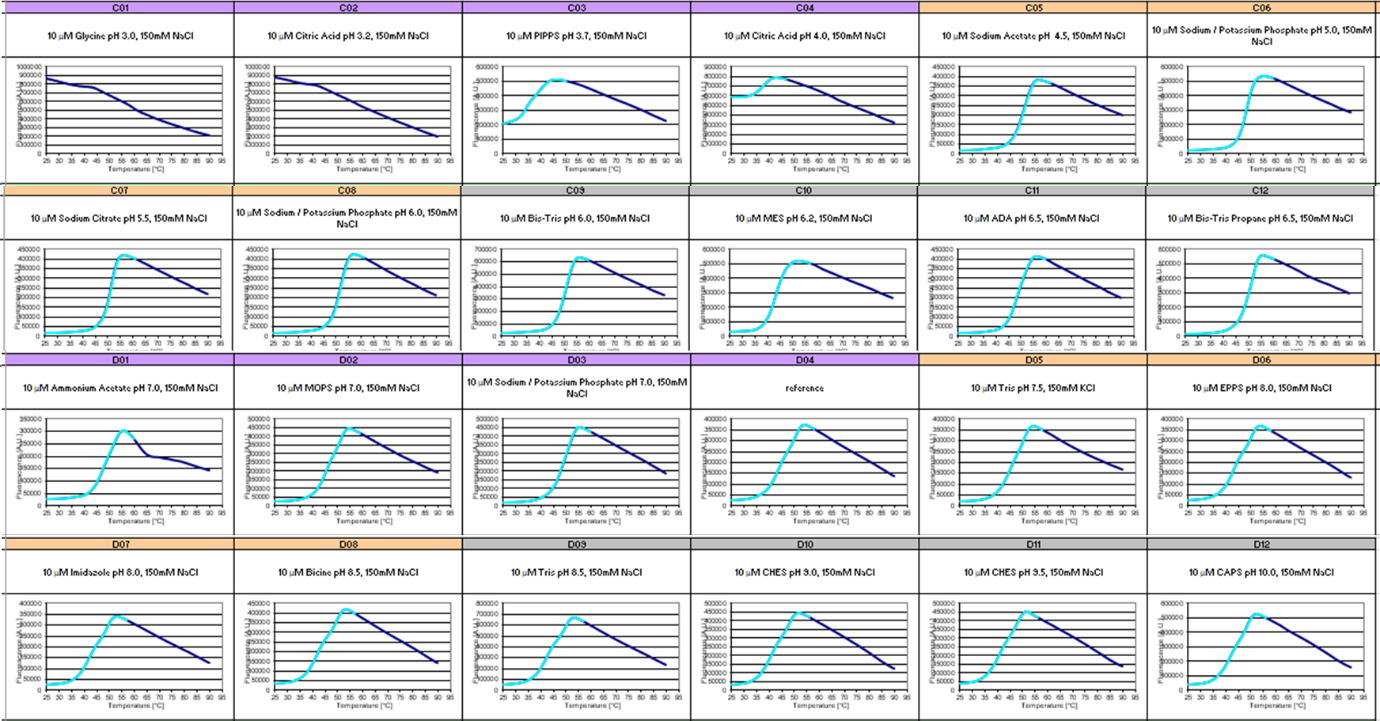


S2 b – curves from well C1 to D12


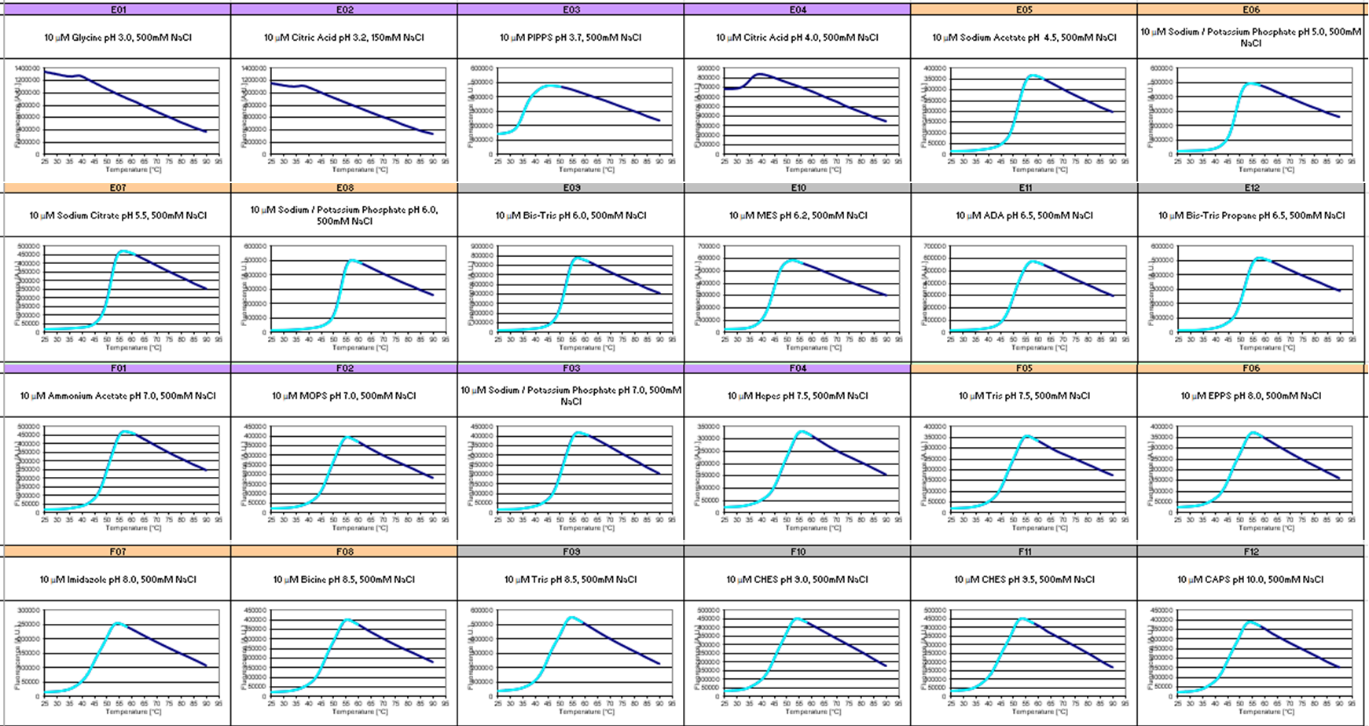


S2 c – Curves from well E1 to F12


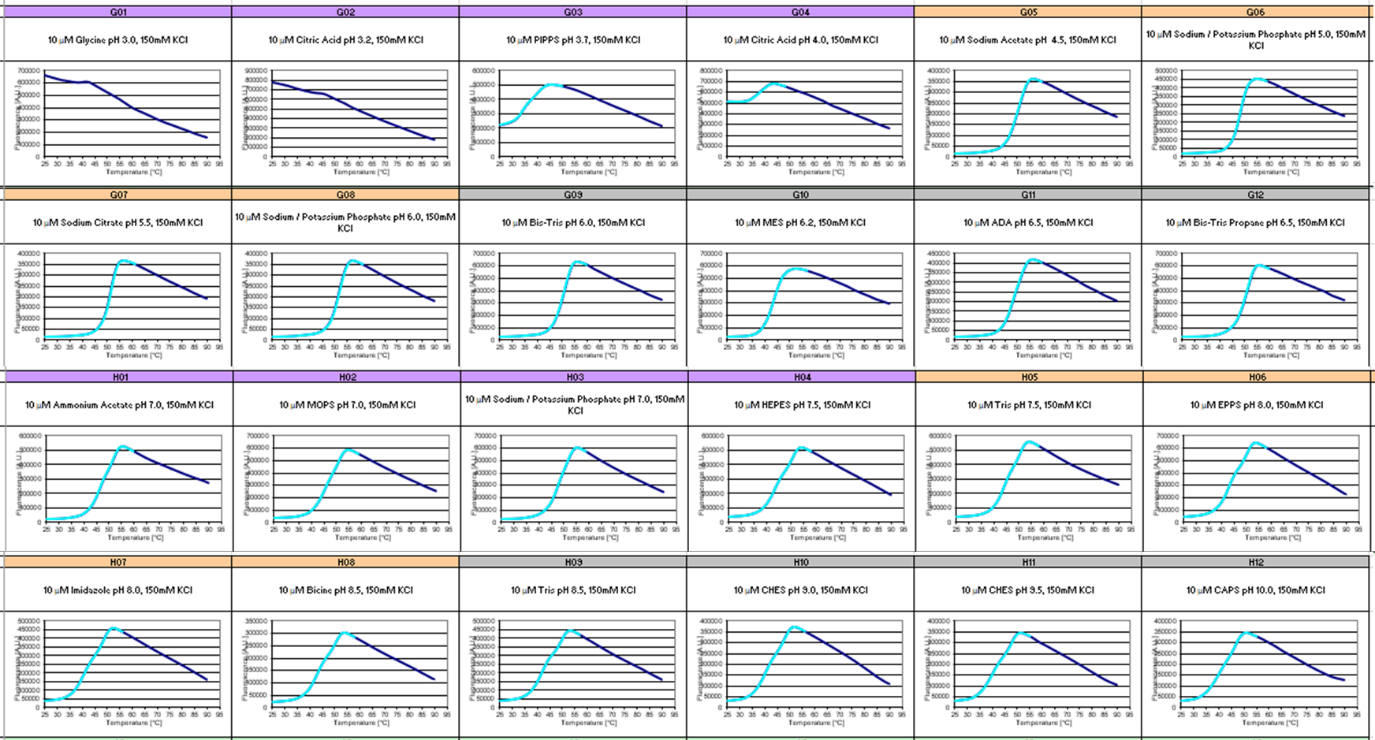


S2 d – curves from well G1 to H12

**
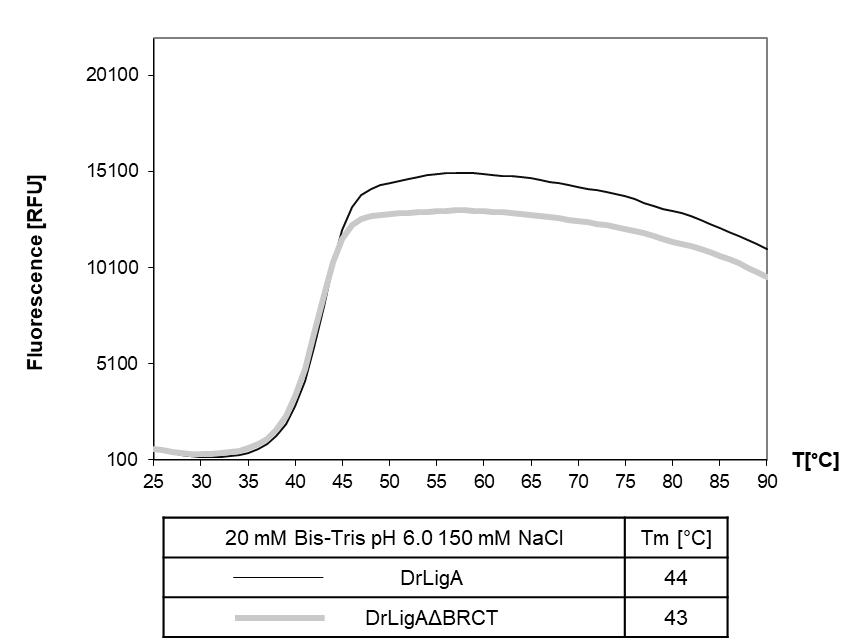
**

**Supplementary Figure S3.** Thermofluor results of DrLigA versus DrLigAΔBRCT: non normalized graphs and melting temperatures.

**
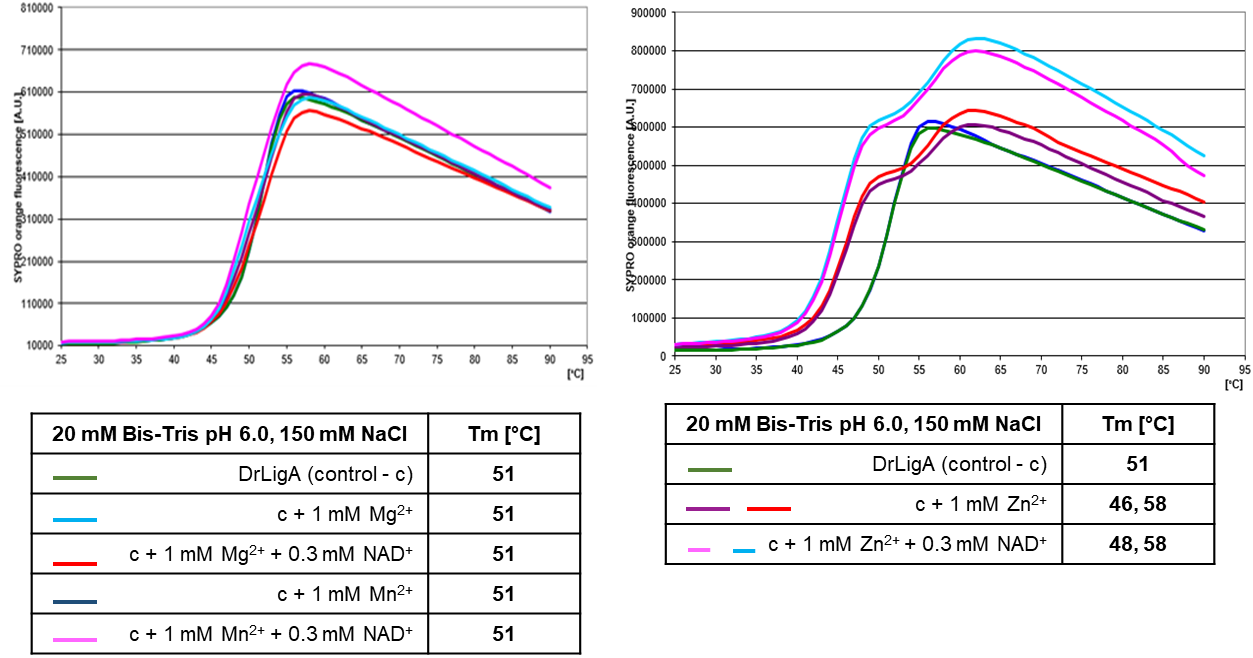
**

**Supplementary Figure S4.** Thermofluor results of DrLigA: non normalized graphs and melting temperatures. Assessment of different additives: 300 µM NAD^+^ with 1 mM Mg^2+^, 1 mM Mn^2+^ or 1 mM Zn^2+^.


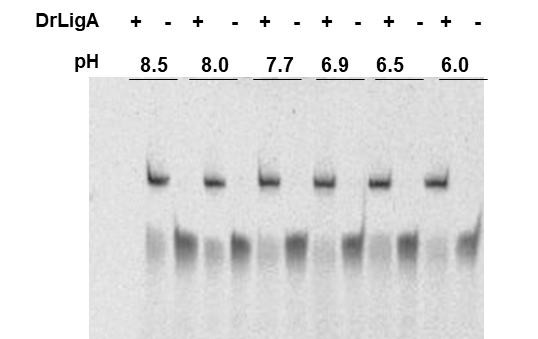


**Supplementary Figure S5.** Gel-based activity assays: DNA ligation by nick closure at different pH. DNA substrate consisted of L1 (FAM-AGGCCATGGCTGATATCGGA), L2 (Phos-TCCGAATTCGAGCT CCGTCG), and L3 (CGACGGAGCTCGAATTCGGATCCGATATCAGCCATGGCCT). The 20 nt substrate is fluorescein (FAM) labelled and contains a phosphate (Phos) in the 5’-end nick. When ligation occurs, a 40 nt product is formed. Symbol + represents added and - absent.


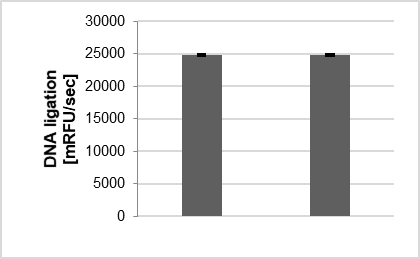


**DrLigA DrLigA∆BRCT**

**Supplementary Figure S6.** Molecular beacon-based activity assay with 330 nM of protein. DNA ligation by nick closure with DrLigA full-length versus truncated DrLigA∆BRCT. The increase in fluorescence was measured as relative fluorescence units (RFU) over time, and the ligation activity is represented as the initial velocity in mRFU per sec (mRFU/sec). Experiments performed at pH 7.0, with 1 mM Mn^2+^ and 5 µM NAD^+^. Error bars represent the standard deviation from the mean of duplicates.


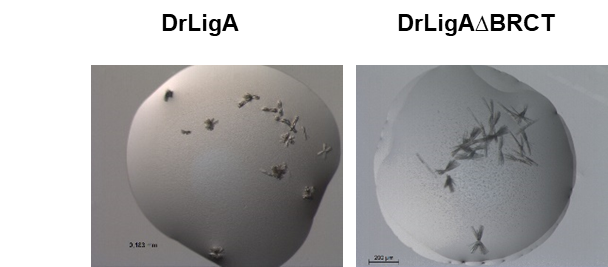

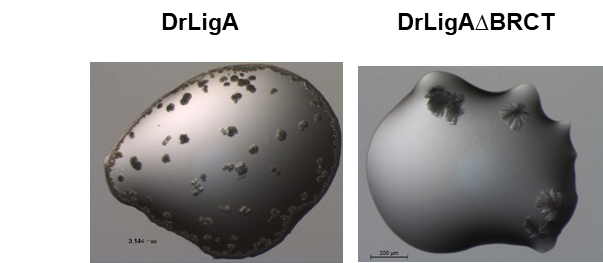


**Supplementary Figure S7.** Crystallization of DrLigA versus DrLigA∆BRCT. On the left, comparison of needle that were obtained in condition A3 of JCSG^+^ screen HT_96 (Molecular Dimensions). On the right, comparison between crystals of DrLigA and DrLigA∆BRCT in condition A8 of Structure Screen 1 & 2 HT_96 (Molecular Dimensions).


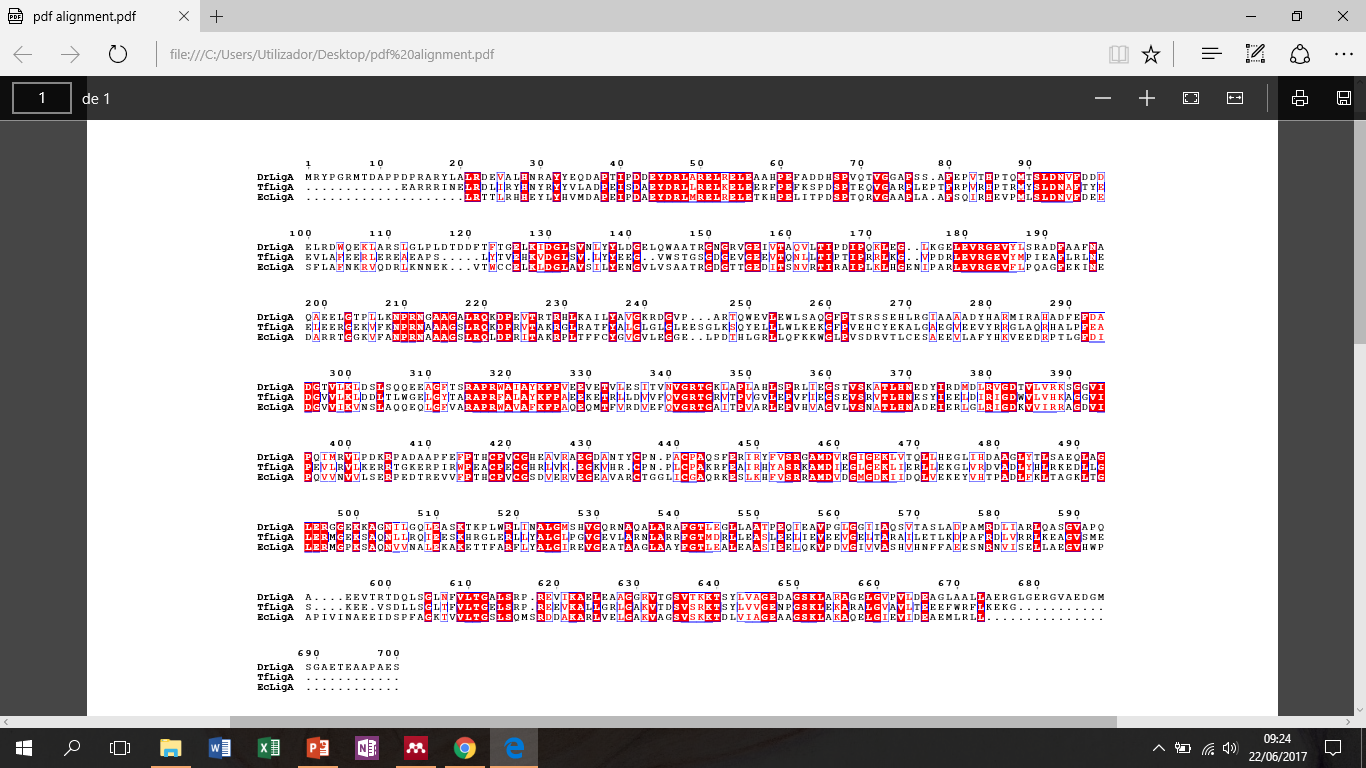


**Supplementary Figure S8.** Multiple sequence alignment of full-length sequence of DrLigA, ThLigA (*T. filiformis*), EcLigA (*E. coli*) performed with ESPript 3.0 (<https://espript.ibcp.fr/ESPript/ESPript/>). Conserved residues are enclosed in red boxes.
